# *HMOX1* Gene Promoter Alleles and High HO-1 Levels Are Associated with Severe Malaria in Gambian Children

**DOI:** 10.1371/journal.ppat.1002579

**Published:** 2012-03-15

**Authors:** Michael Walther, Adam De Caul, Peter Aka, Madi Njie, Alfred Amambua-Ngwa, Brigitte Walther, Irene M. Predazzi, Aubrey Cunnington, Susanne Deininger, Ebako N. Takem, Augustine Ebonyi, Sebastian Weis, Robert Walton, Sarah Rowland-Jones, Giorgio Sirugo, Scott M. Williams, David J. Conway

**Affiliations:** 1 Medical Research Council Laboratories, Fajara, Banjul, Gambia; 2 National Institute of Allergy and Infectious Diseases, National Institutes of Health, Rockville, Maryland, United States of America; 3 Vanderbilt Medical Center, Division of Human Genomics and Center for Human Genetics Research, Nashville, Tennessee, United States of America; 4 Department of Immunology and Infection, Faculty of Infectious and Tropical Diseases, London School of Hygiene & Tropical Medicine, London, United Kingdom; 5 Institute of Medical Microbiology, Immunology and Parasitology, University Clinic Bonn, Bonn, Germany; 6 Department of Internal Medicine, Neurology and Dermatology University of Leipzig, Leipzig, Germany; 7 Institute of Health Sciences Education, Barts and the London School of Medicine & Dentistry, Queen Mary University, London, United Kingdom; 8 Weatherall Institute of Molecular Medicine, John Radcliffe Hospital, Oxford, United Kingdom; 9 Centro di Genetica, Centro di Ricerca Scientifica, Ospedale San Pietro FBF, Rome, Italy; Faculdade de Medicina da Universidade de Lisboa, Portugal

## Abstract

Heme oxygenase 1 (HO-1) is an essential enzyme induced by heme and multiple stimuli associated with critical illness. In humans, polymorphisms in the *HMOX1* gene promoter may influence the magnitude of HO-1 expression. In many diseases including murine malaria, HO-1 induction produces protective anti-inflammatory effects, but observations from patients suggest these may be limited to a narrow range of HO-1 induction, prompting us to investigate the role of HO-1 in malaria infection. In 307 Gambian children with either severe or uncomplicated *P. falciparum* malaria, we characterized the associations of *HMOX1* promoter polymorphisms, *HMOX1* mRNA inducibility, HO-1 protein levels in leucocytes (flow cytometry), and plasma (ELISA) with disease severity. The (GT)_n_ repeat polymorphism in the *HMOX1* promoter was associated with *HMOX1* mRNA expression in white blood cells *in vitro*, and with severe disease and death, while high HO-1 levels were associated with severe disease. Neutrophils were the main HO-1-expressing cells in peripheral blood, and *HMOX1* mRNA expression was upregulated by heme-moieties of lysed erythrocytes. We provide mechanistic evidence that induction of *HMOX1* expression in neutrophils potentiates the respiratory burst, and propose this may be part of the causal pathway explaining the association between short (GT)_n_ repeats and increased disease severity in malaria and other critical illnesses. Our findings suggest a genetic predisposition to higher levels of HO-1 is associated with severe illness, and enhances the neutrophil burst leading to oxidative damage of endothelial cells. These add important information to the discussion about possible therapeutic manipulation of HO-1 in critically ill patients.

## Author Summary

HO-1 is an important anti-inflammatory enzyme induced by several stimuli associated with critical illness. In humans, the amount of HO-1 produced is influenced by a genetic polymorphism in the gene promoter region. Using *Plasmodium falciparum* malaria that can cause a sepsis-like syndrome as an example, we characterize the associations between the (GT)_n_ polymorphism, HO-1 protein levels and *HMOX1*-mRNA expression with severity of malaria in 307 Gambian children. Our results support the functionality of this polymorphism, demonstrate that *P. falciparum* infections increase HO-1 levels, and indicate that a genetic predisposition to strongly upregulate HO-1 is associated with severe forms of malaria and increased risk of dying. We identify neutrophils as the main HO-1-producing blood cells, and provide evidence that hemin-mediated induction of *HMOX1* in neutrophils *in vitro* enhances the oxidative burst. In this way sequestered neutrophils may contribute to oxidative damage of endothelial cells, which may be part of a causal pathway explaining the association between short (GT)_n_ repeats and increased disease severity. Our findings imply that the beneficial effects of HO-1 may be limited to a narrow window of concentrations, which should be born in mind when considering the therapeutic potential of manipulating HO-1 induction in critically ill patients.

## Introduction

Heme oxygenase (HO) is the rate limiting enzyme that catabolizes free heme into carbon monoxide (CO), ferrous iron, and biliverdin/bilirubin [1]. To date, two functional isoforms (HO-1, HO-2) have been described. While HO-2 is constitutively produced by most cells, HO-1 protein is induced by its substrate heme and a broad array of acute stress stimuli, many of which are associated with critical illnesses [2]. HO-1 induction produces cytoprotective and anti-inflammatory effects by reducing intracellular heme availability, through generation of CO and bilirubin, through stimulation of ferritin synthesis [3], and possibly, by heme-independent mechanisms of transcriptional regulation [4]. HO-1 is an essential enzyme in humans and mice; deficiency in humans is deleterious, predominantly affecting endothelial cells and the reticuloendothelial system, and results in a greatly reduced life expectancy [5]. However, much of what is known about HO-1 function is derived from experiments in animal models or in *in vitro* experiments. The impact of *HMOX1* over- or under-expression, silencing or knockout and the concomitant changes in protein levels in a physiological or homeostatic context [6] or in humans [7] is less clear.

The blood stage of malaria infection is characterized by hemolysis and consequent release of hemoglobin and its heme moiety [8]. Elegant mechanistic studies in mice have shown that free heme has a profound pro-inflammatory and cytotoxic effect in malaria, increasing susceptibility to experimental cerebral malaria (ECM), and hepatic failure. These adverse events can be prevented by HO-1 induction or administration of CO that can reduce the levels of free heme [9], [10]. Marked differences amongst mouse strains in the kinetics of HO-1 in response to *P. berghei* ANKA infection appeared to determine susceptibility to ECM, suggesting that regulation of *HMOX1* expression is a crucial factor in this model. However, higher HO-1 levels in the murine liver appeared to allow the development of liver stage parasites by reducing the host inflammatory response, indicating that optimal regulation of HO-1 must balance control of pathogen replication with protection from inflammatory damage during infection [11].

As expected, evidence of increased expression and activity of HO-1 has been observed in human malaria [12], [13], [14], [15], but its functional relevance has been far more difficult to establish. Other than inbred mice, the amount of HO-1 produced in response to a defined stimulus in humans may be influenced by a (GT)_n_ repeat length polymorphism in the promoter region of the *HMOX1* gene [16]. In various chronic inflammatory conditions and other diseases, long *HMOX1* (GT)_n_ repeats, associated with lower HO-1 protein have been identified as disease risk factors [17]. This has led to the hypothesis that the ability to mount a strong HO-1 response is beneficial for people living in malaria endemic areas, and that the disease may have applied selective pressure for shorter (GT)_n_ repeats [17].

The potential role of HO-1 and CO has also been recognized in other critical illnesses [18], and in a murine model free heme clearly contributed to the pathogenesis of severe sepsis [19]. If the protective effect of CO or other products of the enzymatic reaction catalyzed by HO-1 can be established in man, this could provide novel avenues for treatment using therapeutic CO inhalation, or systemic administration of CO releasing molecules (CORM), capable of releasing CO in a controlled fashion [20]. HO-1 has thus moved to “center stage” for a variety of infectious diseases, not just for malaria [20], [21]. A recent report on critically ill patients measuring CO bound hemoglobin (COHb) levels of which 85% can be ascribed to HO-1 mediated heme metabolism [22] indicates that both excessively low or high levels of COHb appear to be associated with death [23]. This indicates that the protective effects of HO-1 are limited to a narrow range of HO-1 concentrations [18].

While HO-1 uses the highly cytotoxic heme as a substrate, one of the products of the enzymatic reaction, ferrous iron, is released into the endoplasmic reticulum (ER). In this form, iron is redox active and can catalyze the formation of organic and inorganic reactive oxygen species (ROS) [24]. HO-1 thus has both anti-oxidant and oxidant properties. In malaria its induction may be particularly enhanced by a pronounced intravascular hemolysis liberating considerable amounts of heme [25] that require degradation by HO-1 in endothelial cells, resulting in an increase of ferrous iron. Indeed, *in vitro* studies indicate that the equimolar production of anti-oxidant bilirubin and ferrous iron by HO-1 results in an overall pro-oxidant effect [26], and that high HO-1 levels can lead to tissue damage [27]. In light of this, it has been hypothesized with regard to the effect of the (GT)_n_ repeat length polymorphism in the *HMOX1* gene promoter that – in contrast to what has been observed for chronic inflammatory conditions - short repeat array alleles may cause susceptibility to severe malaria in humans [28]. Consistent with this, short alleles were found to be associated with risk of cerebral malaria in Myanmar [29], and Angola [30].

The functional duality is problematic with respect to developing adjuvant therapies for severe malaria based on induction of HO-1 or administration of CO, and highlights the need to understand better the regulation and function of HO-1 in humans in relation to both promoter polymorphisms and malaria.

In the present study we characterized in detail the genetic and functional associations between *HMOX1* promoter polymorphisms, HO- 1 inducibility, *HMOX1* expression and severity of malaria in Gambian children exposed to seasonal malaria. We show that short (GT)_n_ repeat alleles in the *HMOX1* gene are associated with higher *HMOX1* expression in white blood cells of this population, and that short repeat alleles are strongly associated with severe disease and death, whilst high *HMOX1* mRNA and HO-1 protein levels are associated with severe disease. We establish that neutrophils are the main HO-1 expressing cell type in peripheral blood *ex vivo*, and demonstrate *in vitro* that *HMOX1* mRNA expression in purified neutrophils can be upregulated further by lysed erythrocytes, or hemin. We provide mechanistic evidence that hemin-mediated *HMOX1* expression potentiates the neutrophil respiratory burst, and propose that this may be part of a causal pathway driving the association between short (GT)_n_ repeats and increased disease severity.

## Materials and Methods

### Ethics statement

The study was reviewed and approved by the Gambian Government/MRC Joint Ethics Committee and the Ethics Committee of the London School of Hygiene & Tropical Medicine (London, UK). Between September 2007 and January 2010, after written informed consent was obtained from the parents or guardians, a total of 153 severe and 154 uncomplicated malaria cases were enrolled. (see Table S1 in Text S1 for detailed information).

### Subject recruitment, study design and study procedures

Subjects enrolled in this study were recruited from an ongoing health centre based study comparing children with uncomplicated and severe malaria disease resident in a restricted peri-urban area of the Gambia described in more detail previously [31]. Uncomplicated malaria (UM) was defined as an episode of fever (temperature >37.5°C) within the last 48 hours with more than 5000 parasites/µl detected by slide microscopy. Severe malaria (SM) was defined using modified WHO criteria [32]: severe anaemia (SA), defined as Hb<6 g/dl; severe respiratory distress (SRD) defined as serum lactate >7 mmol/L; cerebral malaria (CM) defined as a Blantyre coma score ≤2 in the absence of hypoglycaemia or hypovolaemia, with the coma lasting at least for 2 hours; severe prostration (SP) defined as inability to sit unsupported (children>6 months) or inability to suck (children≤6 month). The term “disease severity” refers to comparisons between UM and SM, and, where indicated, to a comparison across disease entities grouped according to increasing severity. To avoid the confounding effects of other pathogens in children with concomitant systemic bacterial infections, children with clinical evidence of infections other than malaria were not enrolled into the study. For some experiments, healthy children (HC, n = 6) of the same age were enrolled as controls. On admission (D0, also referred to as “acute disease”) and after 4 weeks (D28±3 days, also referred to as “convalescence”) one ml of blood was collected in RNA stabilizing agent (PAXgene Blood RNA system, Pre-AnalytiX) and a maximum of 4 mls of blood (mean: 3.2 mls CI 95%: 3.1–3.3 mls) were collected into heparinized vacutainers (BD). Four buccal swabs were performed using sterile mouth brushes (Cytobrush plus, Henley's Medical, UK) and stored in a DNA-stabilizing buffer containing 10 mM Tris, 10 mM EDTA, 0.5% Sarkosyl for subsequent DNA extraction. All patients received standard care according to the Gambian Government Treatment Guidelines, provided by the health centre staff. The children's health was reviewed 7 days after admission. Healthy adult volunteers were bled for the *in vitro* experiments after informed consent was obtained.

*P. falciparum* parasites were identified by slide microscopy of 50 high power fields of a thick film. Full differential blood counts were obtained on days 0 and 28 using a Medonic instrument (Clinical Diagnostics Solutions, Inc).

### Cell preparation

Blood samples were processed within 2 hours of collection. Flow cytometry was performed on 300 µl of whole blood collected into heparinized tubes. HO-1 induction assays were performed on 200 µl of whole blood collected on D28. From the remaining sample, plasma was removed, stored at −80°C and replaced by an equal volume of RPMI 1640 (Sigma-Aldrich). PBMC were isolated after density centrifugation over a 1.077 Nycoprep (Nycomed, Sweden) gradient (800 g, 30 min) and washed twice in RPM 1640. PBMCs were used for other studies [31]. The remaining PBMC deficient blood suspension underwent a further density centrifugation over Histopaque 1119 (Sigma Aldrich) to isolate granulocytes that were used for Western blood analysis of HO-1 expression after a microscopic purity check with Giemsa stain.

### Flow cytometry

Whole blood was incubated for 35 min at 4° in the dark with the following cocktail of surface antibodies: 5 µl each of PE anti-CD16b, Per CP anti-CD14, PE-Cy7 anti-CD4, APC anti-CD19 (all Becton Dickinson), Pacific blue anti-CD3 and 4 ul of APC-AF 750 anti-HLA-DR (both Ebioscience), or a cocktail of manufacturer matched isotype controls. Thereafter, erythrocytes were lysed using FACS lysing buffer (Becton Dickinson), and the remaining cells were fixed and permeabilized (Cytofix/Perm reagent; Becton Dickinson). After a blocking step with 5% of mouse serum (4°C, 15 min) intracellular staining (4°C, 30 min) for HO-1 was performed with 3.5 µl of FITC anti-HO-1 (Abcam). Samples were acquired on a 3 laser/9 channel CyAn ADP flowcytometer and analysed using FlowJo 7.25 (Tree Star Inc.).

### RT-PCR

For quantitative reverse transcription-polymerase chain reaction (qRT-PCR), total RNA was extracted from PAX tubes, collected from study patients following the manufacturer's instructions and reverse transcribed into cDNA using TaqMan reagents for reverse transcription (Applied Biosystems), according to the manufacturer's protocol. In addition, whole blood used for HO-1 induction assays from samples obtained on day 28 were collected into Trizol LS (Invitrogen) and the RNA precipitated by a chloroform/ethanol step. Isolation of RNA from neutrophils or whole blood used in the *in vitro* assays was performed with the RNeasy Mini kit (Qiagen) after collection and storage of the cells into RLT buffer. Gene expression profile for *IL-10* was measured previously on a subset of samples from the clinical study and were used for correlation analysis [31]. *HMOX1* gene expression was determined by qRT-PCR on a DNA Engine Opticon (MJ Research) using the TaqMan Probe kit with primers (all Metabion) as previously published [33]. 18S rRNA, amplified using a commercially available kit (rRNA primers and VIC labeled probe, Applied Biosystems), was assayed as a housekeeping gene with a stable expression profile in this setting regardless of disease severity or time point [31]. Data were analysed using Opticon Monitor 3 analysis software (BioRad) and are expressed as the ratio of the transcript number of the gene of interest over the endogenous control, 18S rRNA.

### HO-1 ELISA

Levels of soluble HO-1 were determined in plasma (1∶50 diluted) or cell culture supernatants (neat) by ELISA in duplicate wells of Immunolon HX4 plates, using the HO-1 human ImmunoSet Kit (Stressgen).

### Histidine rich protein-2 (HRP-2) ELISA

A commercial HRP-2 ELISA kit (CELISA, Cellabs, Australia) was used to quantify HRP-2 in plasma samples diluted 1∶20, in duplicate wells of Immunolon HX4 plates. Some samples were out of range and were repeated at a 1∶2 dilution if below the bottom of the standard curve, or at a 1∶100 dilution if above the top of the standard curve.

### Heme measurement

Free heme in plasma was quantified using a published method [9]. Briefly, plasma was centrifuged at 1000 g for 5 min and the supernatant passed through a Microcon YM-3 column (Millipore, 14,000 g for 100 min at RT) to remove proteins. Free heme from protein depleted plasma and heme content in lysates of infected and uninfected RBC as well as a solution of uninfected intact RBC was quantified by a chromogenic assay (QuantiChrom Heme Assay Kit, BioAssay Systems).

### Western blot

Immunodetection of HO-1 protein in lysates of isolated neutrophils was performed using a protocol adapted from [4]. Polyclonal rabbit anti-HO-1 antibodies obtained from StressGen Biotechnologies Corp. (Victoria, BC, Canada) were used. Polyclonal goat anti-actin (Santa Cruz) antiserum was used for staining as loading controls. Briefly, 10 µg of cell lysate proteins was separated by reducing sodium dodecyl sulphate polyacrylamide gel electrophoresis using precast Nupage gels and MOPs buffer in the X-cell mini electrophoresis chamber (Life Technologies). Separated proteins were then transferred onto methanol treated PVDF membranes using the X-cell mini blotting system. Blotted membranes were rinsed in 1×PBS and blocked overnight at 4°C in blocking buffer containing 5% non-fat milk in 1×PBS and 0.1% Tween 20 (Sigma Aldrich). After the blotted membranes were washed three times in PBS-Tween, they were incubated with constant shaking for 2 h at RT with anti-HO-1, or anti-actin diluted 1∶1,000 in blocking buffer. The membranes were then washed with three changes of PBS-Tween and further probed with horseradish peroxidase-conjugated donkey anti-goat or goat anti-rabbit IgG (Santa Cruz) at a dilution of 1∶10,000 for 2 hours at room temperature with constant shaking. Following three washes in PBS-Tween, membranes were rinsed in 1×PBS and bound antibodies were revealed by chemiluminescent detection performed with the Amersham ECL detection kit according to the manufacturer's instructions.

### Analysis of the *HMOX1* promoter (GT)_n_ repeat length polymorphism

Material from mouth brushes was eluted into transport buffer and incubated with Proteinase K, guanidine hydrochloride, and ammonium acetate (Sigma Aldrich, UK, at final concentrations of 262 µl/ml, 1.57M and 0.59M, respectively), for 1 hr at 60°C. Ice cold chloroform was added to each sample at a ratio of 1 to 1.9 followed by a 5 min centrifugation at 1000 g. The upper layer was transferred onto 10 mls pure ethanol and kept at −20°C for 1 hour to precipitate the DNA. After 15 min centrifugation at 1200 g the pellet was resuspended in 70% ethanol, washed again (1200 g, 5 min), resuspended in 100 µl 1X TE buffer (Sigma Aldrich, UK), and stored at −20°C until processing.

The 5′-flanking region of the *HMOX1* gene containing a (GT)_n_ repeat was amplified by PCR using a fluorescein-conjugated sense primer (*HMOX1*_microsat_ (9/10) 5′-AGAGCCTGCAGCTTCTCAGA- 3′) and an antisense primer HeOP-1/R *HMOX-1*mi (1/3) 5′-ACAAAGTCTGGCCATAGGAC-3′) previously described [29], [34]. Samples that did not amplify with these primers were amplified using a second set of primers; *HMOX-2*micro-Fwd (2/3) 5′ CTTTCTGGAACCTT CTGGGAC 3′ designed according to the published sequence [35] and the above antisense primer. Thirty-five cycles were performed under the following conditions; 96° for 1 minute, 95° for 30 seconds, 60° for 30 seconds, 72° for 3 minutes. The sizes of the microsatellites were determined by the use of a laser based automated DNA sequencer; ABI genetic analyser 3130xl (Applied Biosystems, Forster City, Calif, USA), with a cloned fragment of 28 bp that was used as a size marker. Allele scoring was performed using GeneMapper version 4.0 (Applied Biosystems, Forster City, Calif, USA), by 2 investigators blinded to the disease status of donors. For analysis purposes, alleles were subsequently divided into “S” (for short <27 repeats), “M” (for medium 27 to 32 repeats), and class “L” (for long >32 repeats), using an established classification [33], [34], [36].

### Erythrocyte polymorphisms

To determine the frequency of Glucose 6 Phosphate Dehydrogenase (G6PD) deficiency, genomic DNA was genotyped for SNPs A376G (rs1050829) (G6PD A), G202A (rs1050828) (G6PD A-), and T968C (G6PD A-) [37] that are mutations causing reduced enzyme activity [38]. In order to infer the frequency of individuals with blood group O, rs8176719 was typed to identify the frame shift deletion at this position that encodes the O allele [39].

Genotyping was performed on a Sequenom MassArray platform [40]. For each reaction 20 ng of gDNA was used and each genotype was replicated three times. Sickle cell status was determined by metabisulfite test and the genotype was confirmed by cellulose acetate electrophoresis [31].

### *In vitro* induction of HO-1 in whole blood by heat or hemin

From a subset of participants (12 SM, 20 UM), 200 µl of whole blood collected at D28 were kept for 3 hours at 37°C, at 40°C (water bath), or stimulated with hemin (10 µM, Sigma Aldrich) at room temperature, to determine inducibility of HO-1 mRNA. An additional 6 samples from healthy controls were processed similarly. After the incubation, samples were diluted 1∶1 in RNAse free water, and transferred into Trizol LS reagent (Life Technologies). RNA processing and *HMOX-1* gene expression was carried out as described under qRT-PCR.

### Preparation of *P. falciparum* schizont antigen extract

*P. falciparum* parasites (3D7 clone) were cultured *in vitro* as described [31], and were routinely shown to be mycoplasma free by PCR (Bio Whittaker). Schizont-infected erythrocytes were harvested from synchronized cultures by centrifugation through a Percoll gradient (Sigma-Aldrich). *P. falciparum* schizont extracts (PfSE) was prepared by three rapid freeze-thaw cycles between liquid nitrogen and a 37°C water bath. Lysates of uninfected erythrocytes (uRBC lysate) were prepared in the same way.

### HO-1 inducibility in neutrophils

Neutrophils were isolated from whole blood using CD15 beads (Miltenyi, Germany). The purity of neutrophils was assessed by flow cytometry and found to be 95.4% (95%CI: 93.6% to 97.1%). Neutrophils were cultured for various times either with intact, uninfected red blood cells (intact uRBC, containing 6.6 µM heme), uRBC lysate or PfSE (containing 95.3 µM and 99 µM of heme, respectively), growth medium (GM), or 100 µM hemin (Sigma Aldrich). Cell supernatants were harvested and assayed for HO-1 by ELISA, and cells were collected into RLT buffer and processed for *HMOX1* mRNA as described under qRT PCR.

### Neutrophil oxidative burst

To investigate the impact of hemin-induced HO-1 on the neutrophil respiratory burst, 500 uL whole blood of 4 healthy adult donors was diluted 1∶1 in RPMI (Gibco) and incubated for 18 hours at 37°C, in 5% humidified CO_2_ atmosphere, with different concentrations of hemin (0 to 200 µM). Half of the cells were used to measure hemin-induced induction of HO-1 mRNA. A small aliquot of cells was stained with a neutrophil marker (anti CD15ab labeled with APC, Miltenyi) and the ‘live dead cell stain’ (Invitrogen) to assess the viability of neutrophils by flow cytometry after pre-incubation with hemin. The oxidative burst was measured using a validated flow cytometric assay on the remainder of the cells [41]. Briefly, samples were stimulated by adding PMA (Sigma) to a final concentration ranging from 0 to 1000 nM for another 15 minutes. Thereafter, dihydrorhodamine 123 (DHR 123) (final concentration 5 ug/ml) was added for 5 min. Red cells were lysed (with ammonium chloride lysis buffer) and the remaining cells were stained with anti CD15 APC (Miltenyi) and the median fluorescence intensity of rhodamine, the fluorescent oxidation product of DHR 123, was measured in CD15^+^ cells by flow cytometry.

In a separate series of experiments 500 µl of whole blood from another 4 healthy adult donors was incubated with 0–200 µM hemin, either with or without addition of tin protoporhyrin IX dichloride (SnPP; final concentration 10 µM), a non-substrate inhibitor of HO-1 activity [42]. A viability check was performed as described above, and the oxidative burst was induced by stimulation with PMA at a final concentration of 100 nM for 15 min, and the burst was measured as described above.

### Statistical analysis

Flow cytometric results, *HMOX1* mRNA and HO-1 plasma levels obtained on D0 and D28 were compared using linear regression based on ranks, with a random effect to allow for repeated measurements over time. Significance (measured at the 5% level) tests for the effects of malaria group (SM, UM), time (D0 and D28) and their interaction were adjusted for the possible confounding effects of age, gender, duration of prior symptoms and Hb levels, as indicated. Further adjustment for neutrophils was performed for the analysis of WBC, and HO-1 mRNA. Where there was no significant malaria group and time interaction, p-values for the overall comparison of D0 vs D28 are given. Comparisons of SM vs UM are given for each time point separately if the malaria group and time interactions were significant. To allow for the multiple tests resulting from multiple responses and multiple comparisons within a response performed in the model, a false discovery rate (FDR) of 5% was assumed. Using the Benjamini and Hochberg approach [43] only tests with a p-value below 0.012 have an FDR of ≤5%.

Comparison of *HMOX1* mRNA and HO-1 plasma levels between different disease entities during acute disease was performed using linear regression based on ranks, adjusting for the confounding effects of age, gender, duration of symptoms, and for neutrophil counts and Hb levels where indicated.

A multinomial logistic regression model was employed to explore the association between the exposure ‘L allele containing genotype’ and the outcome of different disease entities. Pearson Chi-squared tests were used to compare proportions amongst more than 2 groups. The magnitude of the differences of the long L allele frequencies reported for African, Asian and European populations was explored with fixation (*F*_ST_) indices, using FSTAT [44].

For the analysis of *in vitro* induction of *HMOX1* mRNA in neutrophils, pairwise comparisons using Wilcoxon matched pairs test were performed, with p values adjusted for multiple comparisons using Holm's step down procedure. Where more than two groups were compared, non-parametric one way ANOVA (Friedman test for paired samples, Kruskal Wallis test for unpaired samples) was used with Dunn's post test adjustment for multiple comparisons.

Linear regression was performed to assess whether the magnitude of the oxidative burst induced by a given concentration of PMA increases with increasing concentrations of hemin used for pre-incubation. Analyses were performed using Stata version 10, and Graph Pad PRISM version 5.01.

## Results

In total, 154 uncomplicated (UM), and 153 severe malaria (SM) cases were recruited into this study. The proportions followed up at day 28 were 85.7% for the UM group and 83% for the SM group. Ten children (6.5%) in the SM group died, and an additional 15 (9.8%) were either lost to follow up after hospital discharge or withdrew consent. Nine of the ten deaths occurred in children who were classified as having severe respiratory distress (SRD), defined as lactic acidosis, that has been recognized as the single most important determinant of mortality in severe malaria [45]. Children suffering simultaneously from SRD plus cerebral malaria (CM) had the highest mortality (29.4%), followed by cases with SRD (16.7%). This is consistent with a large study from Kenya, demonstrating that mortality decreases in the following order for different disease entities: [SRD+CM+severe anaemia (SA)]>[SRD+CM]>[SRD]>[SRD+SA]>[CM]>[CM+SA]>[SA]>[severe prostration (SP)] [46]. Where appropriate, cases were grouped according to disease entities and analyzed in the order of increasing severity (Table S1 in Text S1).

### Neutrophils are the main HO-1 expressing cell type in peripheral blood

**Figure 1:**
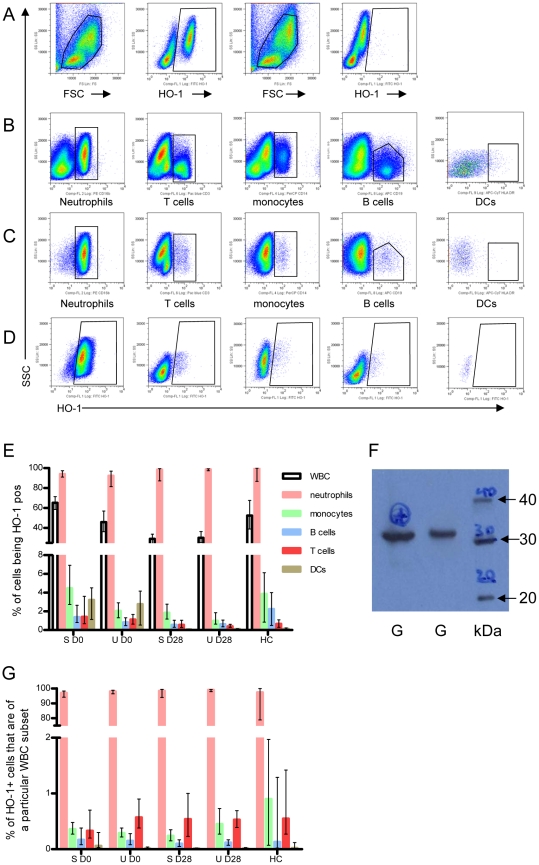
Neutrophils are the main HO-1 expressing cell type in peripheral blood. Whole blood from 16 SM and 21 UM cases was stained for A) intracellular HO-1 expression [plots 3 and 4 in the first row show the isotype control], and B) for lineage markers defining neutrophils (CD16b), T cells (CD3), monocytes (CD14), and B cells (CD19). Dendritic cells were defined as lineage marker negative HLA-DR pos cells. C) HO-1 positive cells were displayed according to expression of lineage markers. D) Neutrophils, T cells, monocytes, B cells and DCs were displayed according to HO-1 expression. The Y axis in all plots is the side scatter (SSC). E) Proportion of white blood cell subsets staining for HO-1. F) representative Western blot showing HO-1 immunoreactive protein in purified granulocytes [G = granulocytes, kDa = molecular weight maker] G) Percentage of HO-1 pos cells according to white blood cell subsets. S = severe; U = uncomplicated cases; HC = healthy controls; D0 = day 0; D28 = day 28. The median with 95% CI is shown for 16 severe, 21 uncomplicated cases and 6 healthy controls.

To identify which leucocyte subsets express HO-1 protein, whole blood collected on days 0 and 28 from 16 SM and 21 UM cases was stained for lineage markers and intracellular HO-1 (Figure 1A–D). In SM, both the proportion and the total number of white blood cells (WBC) expressing HO-1 was 2.2 and 1.3 fold higher during the acute phase compared to convalescence (both with p<0.0001, both adjusted and unadjusted). For UM cases, a smaller difference was observed between time-points that became non-significant after adjustment for percentage (number) of neutrophils. (%WBC UM: p = 0.280 [unadjusted: p<0.0001]; WBC number UM: p = 0.31 [unadjusted: p = 0.01]).

Both the percentage and total number of WBC expressing HO-1 were significantly higher in SM than UM cases on day 0 (% WBC: p = 0.004 [unadjusted: p = 0.001]; total WBC count: p = 0.009 [unadjusted p = 0.008]), while no differences were observed on day 28. Almost all neutrophils stained positive for HO-1, both on D0 (median: 93%, CI 95%: 90.5–96%) and D28 (median: 97%, CI95%: 97–99%), with no difference between SM and UM. Similarly, a median of 98% (CI 95%: 86–99.8%) of neutrophils from healthy controls (HC) stained positive for HO-1 (Figure 1E). Western blot of isolated neutrophils from 4 cases confirmed the presence of HO-1 in purified cells (Figure 1F). Monocytes, B cells, T cells and DCs also expressed HO-1, albeit at lower levels, rarely exceeding 4% of the lymphocyte subset (Figure 1E). In both SM and UM cases the proportions of monocytes, T cells and DCs expressing HO-1 were slightly but significantly higher on D0 compared to D28 (p = 0.002 [unadjusted: p<0.0001], <0.0001 [unadjusted: p<0.0001], <0.0001 [unadjusted: p<0.0001], respectively; Figure 1E). The total number of HO-1 positive neutrophils, monocytes and DCs was significantly higher on Day 0 compared to D28 for both SM and UM cases (p<0.0001 [unadjusted: p<0.0001], p = 0.005, [unadjusted: p = 0.001], p<0.0001[unadjusted: p<0.0001], respectively; Figure S1).

Irrespective of disease status (SM, UM, HC) or time of sampling, a median of 98% (CI95%: 97.3–98.6%) of HO-1 expressing cells were neutrophils, whereas the other cell subsets accounted for less than 1% of HO-1 producing cells in peripheral blood (Figure 1G).

### *HMOX1* mRNA expression in whole blood cells and plasma levels of HO-1 are elevated during acute malaria

**Figure 2:**
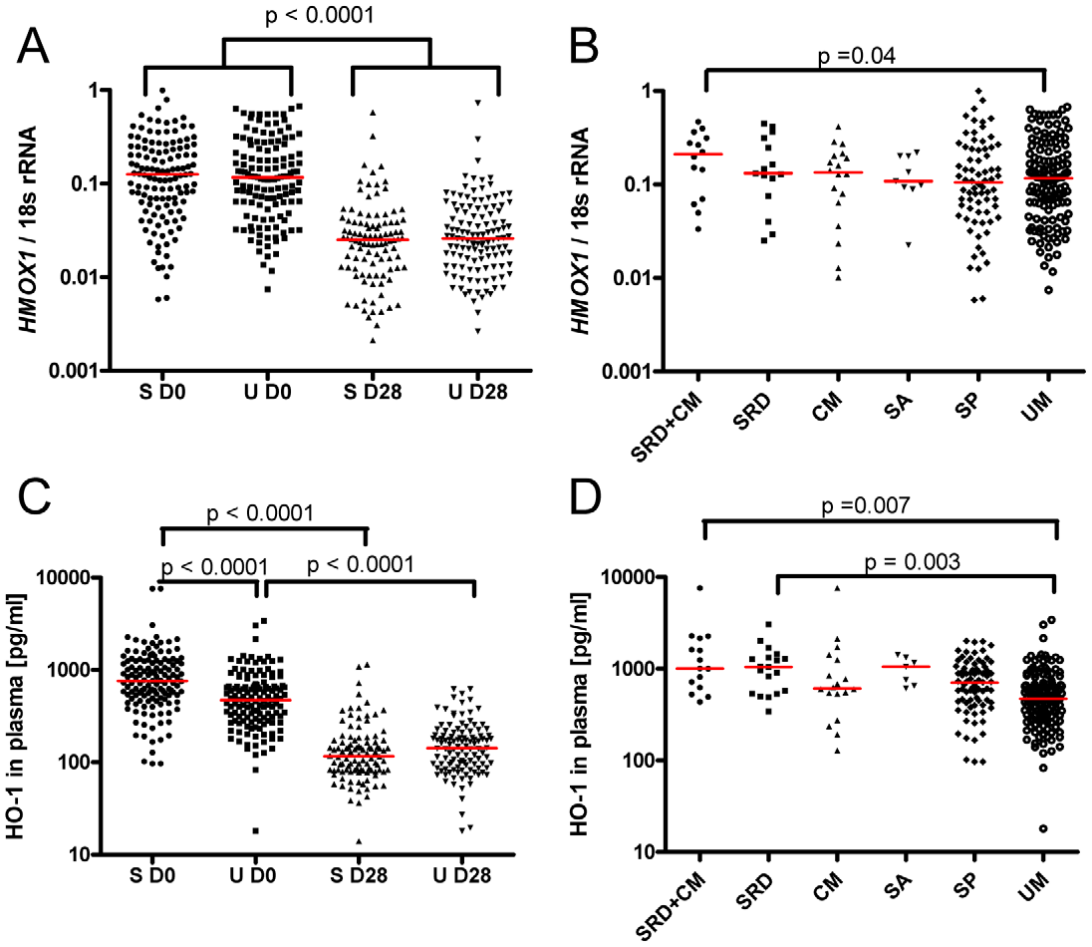
*HMOX1* mRNA and HO-1 protein are upregulated in acute malaria, being highest in cases with SRD. *HMOX1* mRNA levels measured in whole blood are shown comparing A) severe (n = 128) and uncomplicated (n = 134) cases over time, and B) different disease entities on day 0. HO-1 plasma concentrations are shown comparing C) severe (n = 138) and uncomplicated (n = 137) cases over time, and D) different disease entities on day 0. P values shown in A) and C) are derived from the random effects regression model, adjusting for age, gender, duration of symptoms and neutrophil counts. P values shown in B) and D) are from a linear regression model adjusting for age, gender, duration of symptoms, neutrophil counts and Hb. S = severe, U = uncomplicated, D0 = day 0, D28 = day 28. SRD = severe respiratory distress, CM = cerebral malaria, SA = severe anaemia, SP = severe prostration, UM = uncomplicated malaria. The red lines represent the medians.

RNA was extracted from 128 SM and 134 UM cases at both D0 and D28 from blood collected into PAX tubes to assess *HMOX1* mRNA levels by qRT-PCR. Considering that neutrophils were the major source of HO-1 in peripheral blood and that their numbers are slightly higher in acute malaria compared to convalescence [31], the random effects model (from which the p values are derived) additionally adjusted for neutrophil counts to rule out that the observed difference merely reflects different numbers of HO-1 producing neutrophils. For both SM and UM cases a geometric mean 4.3 and 3.7 fold higher *HMOX1* mRNA/18s rRNA ratio was found during acute disease compared to convalescence (p<0.0001 [unadjusted: p<0.0001], for both SM and UM, Figure 2A), while no significant difference was observed between SM and UM on D0 or D28.

When *HMOX1* mRNA expression of peripheral blood cells was assessed for different disease entities during acute disease (Figure 2B), linear regression with UM as a baseline group adjusting for age, gender duration of symptoms and neutrophil counts revealed a trend towards higher levels with increasing disease severity. This reached borderline significance for children classified as having SRD plus CM (p = 0.04) [unadjusted: p = 0.19]. In the UM, SP, SA, CM and SRD groups *HMOX1* mRNA levels at D0 were elevated similarly (3.1 to 4.8 fold higher [geometric means] than on D28, p = 0.36, for comparison among groups, Kruskal Wallis test), but a significantly higher, 10.1 fold increase was measured for the SRD+CM group (p<0.05 compared to UM, Dunn's post test, adjusting for multiple comparisons).

Considering that previous studies report increased HO-1 protein in plasma of critically ill patients, [47], we measured HO-1 levels in plasma on days 0 and 28 for 138 SM and 137 UM cases. Similar low levels of HO-1 were observed during convalescence for both SM and UM, but HO-1 concentrations measured during acute disease (D0) were 5.7 fold (SM) and 3.3 fold (UM) higher than on D28 (p<0.0001 for both groups [unadjusted: p<0.0001 for both groups], Figure 2C), with HO-1 levels in SM being significantly higher than in UM at D0 (p<0.0001) [unadjusted: p<0.0001], but not day 28. Using uncomplicated cases as the baseline group, plasma concentrations of HO-1 measured on day 0 were significantly associated with disease severity in a linear regression model based on ranks adjusting for age, gender, and duration of symptoms (p<0.0001 [unadjusted: p<0.0001]). In particular, patients with SA, SRD and SRD plus CM had significantly higher HO-1 plasma concentrations (SA: p = 0.004 [unadjusted: p = 0.001], SRD: p<0.0001 [unadjusted: p<0.0001], and SRD+CM: p = 0.001 unadjusted: p<0.0001]). After additional adjustment for Hb concentration the overall association between severity and HO-1 levels remained significant (p = 0.004). However, while HO-1 remained significantly elevated in cases with SRD (p = 0.003) and SRD plus CM (p = 0.007), the difference previously seen in the SA group was lost (p = 0.12; Figure 2D). While the origin of plasma HO-1 remains unclear, the latter observation supports the hypothesis that it is derived at least in part from damaged tissues [47]. By definition, SA cases have lower Hb levels, and a higher degree of hemolysis, which is associated with considerable damage of endothelial cells due to release of iron and heme-containing moieties from hemolysed RBC [48]. Adjusting for Hb may even out the effect of hemolysis-driven damage of endothelial cells that may lead to HO-1 release into plasma.

However, additional factors may be responsible for the high HO-1 levels found in patients with SRD, where a significantly higher increase of HO-1 plasma levels was observed between D0 and D28 in patients with SRD or SRD plus CM compared to other entities (p<0.0001, Kruskal Wallis test). While patients with SP, SA or CM had 4.8, 7.7 and 5.5 fold higher values on D0 compared to D28, a 10.2 and 8.9 fold difference between D0 and D28 was measured for patients with SRD plus CM or SRD, respectively. This was significantly higher than the 3.3 fold increase observed for UM (p<0.05, Dunn's post test).

In summary, the data indicate that HO-1 production is induced during acute malaria in peripheral blood cells and probably in various other tissues, and the effect is greatest in cases with SRD.

Interestingly, for both SM and UM, HO-1 levels in plasma correlated well with indirect bilirubin, one of the end products of the reaction catalysed by HO-1, that is usually seen as an indirect measure of HO-1 activity, and has been established as a marker for disease severity [49] (r: 0.69, p<0.0001 [SM] and r: 0.77, p = 0.0012 [UM]; Figure S2).

### Lysates of infected and uninfected RBCs can induce *HMOX1* expression in neutrophils *in vitro*

**Figure 3:**
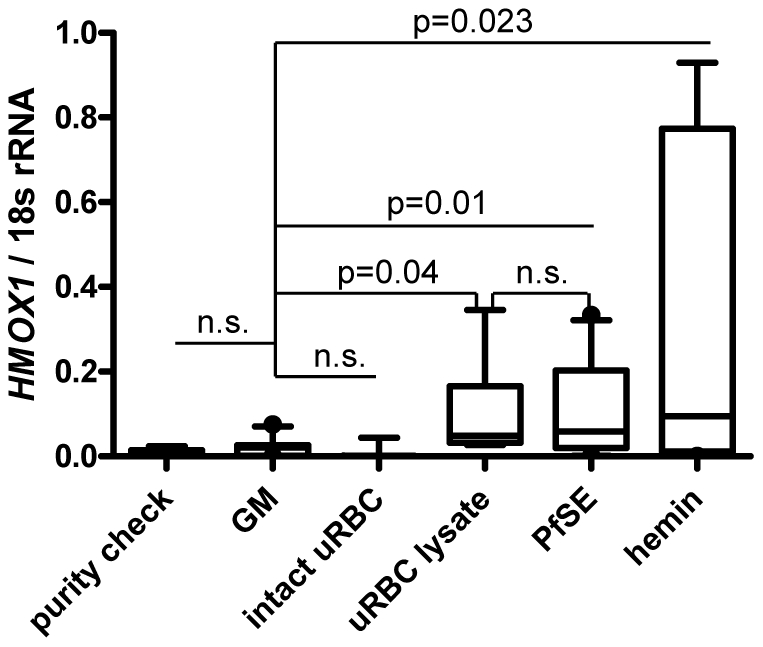
Hemin and lysed RBC can induce *HMOX1* mRNA in neutrophils. Induction of *HMOX1* mRNA in bead-isolated neutrophils is shown in response to 3 hours stimulation with growth medium (GM), intact uninfected RBC, a lysate of uninfected RBC, *P. falciparum* Schizont extract (PfSE), or hemin. Experiments were repeated independently 7 times. Wilcoxon matched pairs test was performed with p-values adjusted for multiple comparisons using Holm step down procedure. Box plots show medians and interquartile ranges.

Considering that parasitaemia [%] correlated with HO-1 in plasma (r: 0.5, p<0.0001) and the observation that the majority of neutrophils contained HO-1, we investigated whether encounter with *P. falciparum* antigens can induce *HMOX1* expression in neutrophils. To this end, *HMOX1* mRNA expression was determined by qRT-PCR in neutrophils purified from whole blood of 7 donors using magnetic beads and cultured for 3 hours with either growth medium (GM) (negative control), 100 µM hemin (positive control), intact uninfected red blood cells (uRBC) at 1×10^8^/ml, or freeze – thaw lysates of either uRBC or *P. falciparum* Schizont extract (PfSE) at a concentration equivalent to 1×10^8^ cells/ml. Heme concentrations were measured in all RBC preparations and were found to be 6.6 µM (intact uRBC), 95.3 µM (uRBC lysate) or 99 µM (PfSE). Culture with lysates of both uRBCs and PfSE resulted in a significant increase in median *HMOX1* mRNA expression compared to culture in GM (2.3 and 2.7 fold with uRBC (p = 0.04) and PfSE (p = 0.01) lysate, respectively), while *HMOX1* mRNA remained at baseline levels in neutrophils cultured with intact uRBCs (Figure 3). Culture in the presence of hemin led to a significant, 4.4 fold median increase in *HMOX1* mRNA in neutrophils (p = 0.023; all results adjusted for multiple comparisons).

To evaluate whether neutrophils could contribute to plasma HO-1 levels by releasing HO-1, we cultured bead-purified neutrophils from an additional 4 donors using the above described conditions for 3, 6, 12, 24 and 36 hours, respectively, and tested the supernatants for HO-1 protein by ELISA. RNA was isolated from neutrophils for determination of *HMOX1* mRNA by qRT-PCR. Although *HMOX1* mRNA expression increased up to 6 hrs in response to uRBC, PfSE and hemin no significant amount of HO-1 could be measured in the supernatants for any of the conditions tested (data not shown).

Taken together, these data demonstrate that *HMOX1* mRNA can be induced in neutrophils in response to hemin as well as RBC lysates containing significant amounts of heme, and suggest that heme released during RBC lysis rather than parasite-derived molecules contribute to this increase. Further, we demonstrate that neutrophils do not release HO-1 in response to these stimuli within 36 hours, and thus are unlikely to contribute to plasma HO-1.

In order to explore a correlation between free heme and HO-1 in plasma from our clinical samples, we attempted to quantify non-protein bound (free) heme. After filtration of the samples heme concentrations were barely measurable (median 1.42 µM, CI95%: 1.37–1.48 µM), being 4.6 fold lower than that in washed preparations of intact uninfected RBCs. We therefore excluded these data from further analysis. However, both RBC counts and Hb levels that may be regarded as surrogates for the degree of hemolysis, and therefore free heme in acute malaria, showed a negative correlation with plasma HO-1 (r = −0.34, p<0.0001 in both cases).

### Hemin-induced *HMOX1* mRNA expression primes the oxidative burst of neutrophils

**Figure 4:**
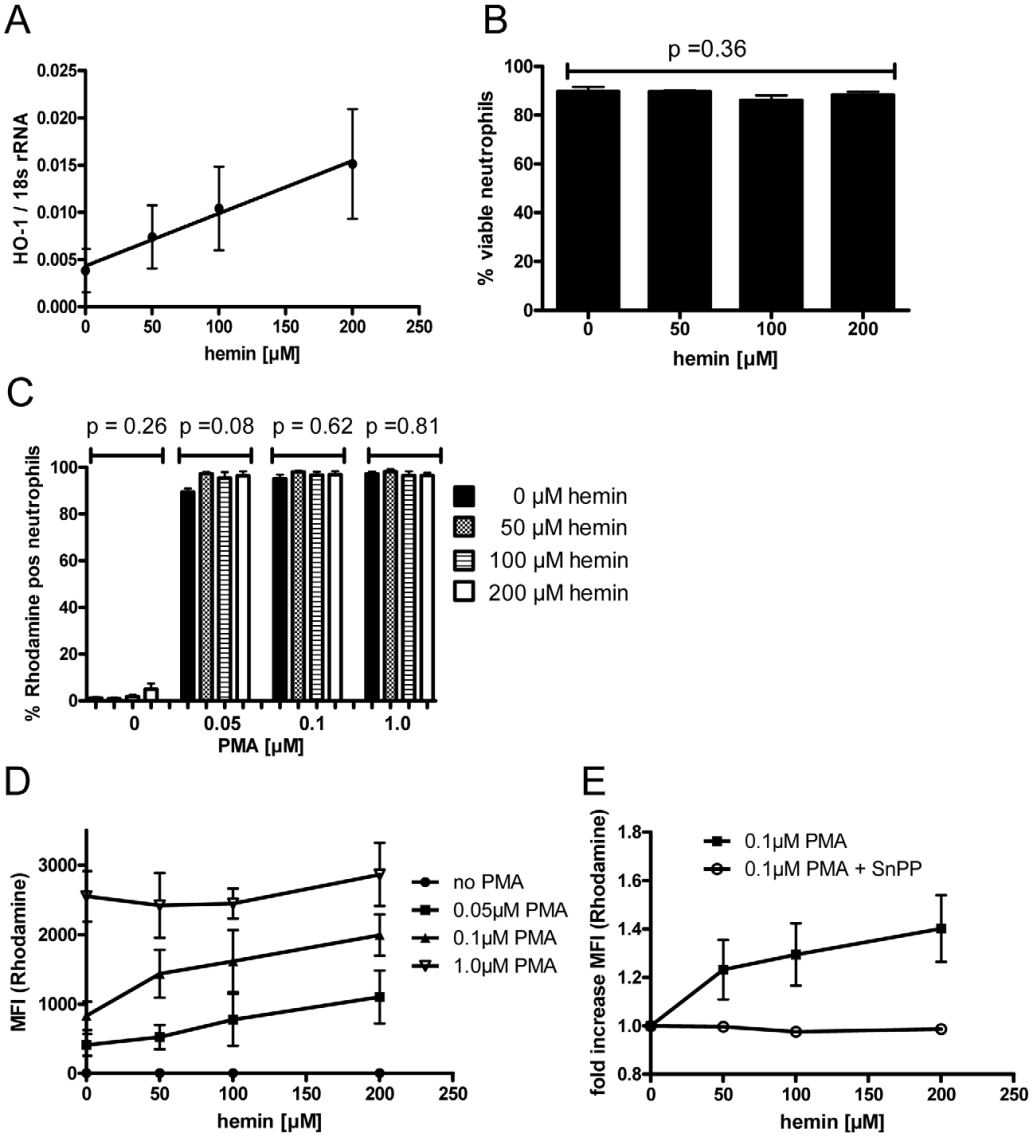
Hemin-induced *HMOX1* induction primes the oxidative burst of neutrophils. In 8 independent experiments, whole blood from different donors was incubated for 18 hours with increasing amounts of hemin in the presence or absence of 10 µM SnPP and the neutrophil oxidative burst induced by PMA was quantified by flow cytometric assessment of oxidation of dihydrorhodamine 123 into the fluorescent rhodamine. A) *HMOX1* mRNA levels induced by various concentrations of hemin. B) Viability of neutrophils after 18 hrs pre-incubation with 0–200 µM hemin. C) Proportion of neutrophils that mount an oxidative burst, defined as positive rhodamine fluorescence. D) Magnitude of the oxidative burst in neutrophils in response to stimulation with 0–1 µM PMA after 18 hrs pre-incubation with 0–200 µM hemin. E) Magnitude of the oxidative burst in neutrophils in response to 0.1 µM PMA after 18 hrs pre-incubation with 0–200 µM hemin with or without addition of 10 µM SnPP. The mean and SE are shown. MFI: median fluorescence intensity.

The observation that *HMOX1* expression can be increased in neutrophils in response to heme prompted us to investigate the role of HO-1 for the neutrophil respiratory burst. The oxidative burst in neutrophils is essential for the host's ability to kill ingested microorganisms and parasites [50], but intense oxidative stress has also been associated with severe forms of malaria, triggering unspecific tissue damage [10]. To assess the impact of hemin-mediated induction of HO-1 on the neutrophil function, the respiratory burst in response to PMA stimulation (0, 0.05, 0.1 and 1 µM) was measured using a validated flow cytometric whole blood assay [41] after overnight incubation of blood from 4 healthy donors with several concentrations of hemin. As expected, pre-incubation with hemin induced *HMOX1* mRNA expression in a dose dependent manner (r^2^_lin regression_ = 0.9, p = 0.0052, Figure 4A), and did not significantly affect the viability of neutrophils (Figure 4B). While hemin pre-incubation alone did not induce the oxidative burst, stimulation with 0.05, (0.1) or [1.0] µM PMA reliably induced an oxidative burst in 94.4%, 96.5% and 96.9% of neutrophils, respectively (p = 0.052, paired measures ANOVA). For each PMA concentration, pre-incubation with different hemin concentrations did not affect the proportion of neutrophils responding with an oxidative burst (Figure 4C). However, the magnitude of the oxidative burst induced with 0.05 µM and 0.1 µM PMA in neutrophils increased significantly with increasing concentrations of hemin used during the pre-incubation (r^2^_lin regression_ 0.92 [0.05 µM PMA] and 0.84 [0.1 µM PMA], with p = 0.037 [0.05 µM PMA] and 0.04 [0.1 µM PMA], Figure 4D). When 1 µM PMA was used, the burst could be maximally stimulated without preincubation with heme. This suggests that hemin-mediated induction of HO-1 may prime neutrophils to mount a stronger oxidative burst.

To further investigate whether the observed effect is mediated by hemin-induced HO-1, we repeated the experiment in the presence and absence of 10 µM tin protoporphyrin (SnPP), an inhibitor of HO-1 activity, using whole blood from another 4 healthy volunteers in separate experiments. At this concentration, SnPP did not significantly affect neutrophil viability compared to pre-incubation with hemin alone (p = 0.125 [0 µM hemin], 1.0 [0.05 µM hemin], 0.125 [0.1 µM hemin] and 0.625 [1 µM hemin], Wilcoxon signed rank test). Further, the addition of 10 µM SnPP did not affect the proportion of neutrophils responding with an oxidative burst to stimulation with 0.1 µM PMA, compared to pre-incubation with hemin alone (p = 0.375 [0 µM hemin], 0.25 [0.05 µM hemin], 0.875 [0.1 µM hemin] and 0.625 [1 µM hemin], Wilcoxon signed rank test). However, hemin pre-incubation in the presence of 10 µM SnPP abrogated the hemin dose-dependent increase of the oxidative burst (r^2^_lin regression_ 0.88, p = 0.035 [no inhibitor] and r^2^ = 0.22, p = 0.23 [with inhibitor], Figure 4E). Taken together, the data indicate that heme-mediated induction of HO-1 primes the oxidative burst in neutrophils.

### Other factors that may induce HO-1

Apart from the availability of its substrate heme, other factors may induce HO-1. In animal models [51], and human hepatoma cell lines [52] HO-1 could be induced by heat exposure. However, in human alveolar macrophages or erythroblastic cell lines [53], as well as in PBMC [54] thermal stress failed to induce HO-1. Since we observed a weak but significant positive correlation between temperature on admission to the clinic and HO-1 plasma levels (r: 0.266, p<0.0001), we explored whether a temperature of 40°C maintained over 3 hours would induce *HMOX1* mRNA in human whole blood, using samples collected on D28 from 12 SM, 20 UM cases and 6 HC. Samples kept at 37°C or cultured with hemin served as negative and positive controls, respectively.

**Figure 5:**
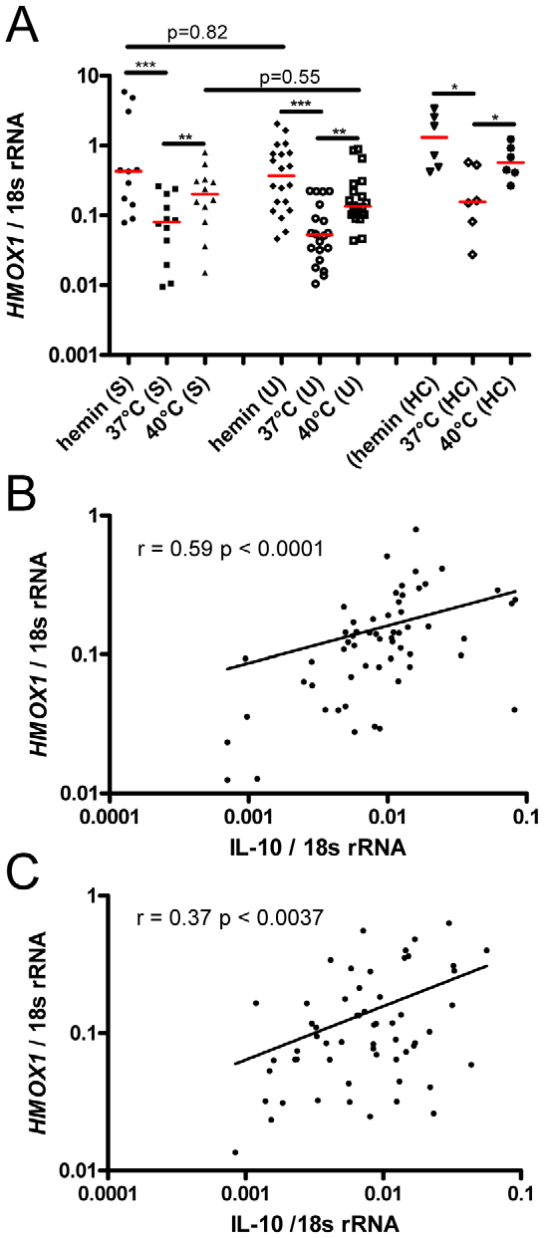
Other factors associated with *HMOX1* mRNA expression. A) *HMOX1* mRNA was measured in whole blood from convalescent SM (n = 12) and UM (n = 14) cases and healthy controls (HC, n = 6). For 3 hours, blood was kept in a water bath at 37°C, 40°C or stimulated at room temperature with hemin. Measurements within each group (SM, UM, HC) were compared using the Friedman test, (a non-parametric ANOVA for repeated measures) with Dunn's post-test adjustment for multiple comparisons. Differences between SM and UM for hemin or heat induction were assessed using the Mann Whitney test. The red lines indicate the medians. * p≤0.05, ** p≤0.005, *** p≤0.0005. mRNA levels for *HMOX1* and IL-10 measured on day 0 were correlated for B) 58 SM [r: 0.59, p<0.0001] and C) 59 UM cases [r: 0.37, p = 0.0037].

In all three groups (SM, UM, HC) both incubation at 40° as well as with hemin resulted in a significant upregulation of *HMOX1* mRNA compared to cells kept at 37°C, with no significant differences observed between groups (Figure 5A). In a separate experiment using blood from 5 healthy donors we verified that incubation at 40°C for 3 hours did not lead to a significant change in hemolysis markers such as heme, haptoglobin or LDH, compared to incubation at 37°C (data not shown).

For murine macrophages, IL-10 has been shown to induce HO-1 [55]. We therefore correlated *HMOX1* mRNA to IL-10 mRNA from D0 samples for 58 SM and 59 UM cases for which IL-10 mRNA measurements were available from a previously reported study [31]. For both SM and UM a positive correlation (SM: r = 0.59, p<0.0001; UM: r = 0.37, p = 0.0037) was found in whole blood, compatible with a role for IL-10 as an inducer of HO-1 in human blood cells (Figure 5B, C).

### *HMOX1* promoter genotypes without L-allele are associated with SRD and mortality

The extent to which *HMOX1* is upregulated in an individual in response to a defined stimulus may be influenced by genetic polymorphisms in the promoter region of the *HMOX1* gene of which several have been described, (reviewed by [16]). Of particular interest, a (GT)_n_ repeat length polymorphism regulates the promoter activity and gene expression, with short repeats (<27 repeats) resulting in an increased transcription of *HMOX-1* compared to alleles with long repeats (>32 repeats) [33], [56]. Associations of the (GT)_n_ polymorphism with disease outcomes have been explored in numerous association studies for various diseases, recently reviewed in [17]. To investigate whether the (GT)_n_ polymorphism is associated with disease severity in malaria, we genotyped this microsatellite for 142 SM and 151 UM cases for whom DNA samples were available.

**Figure 6:**
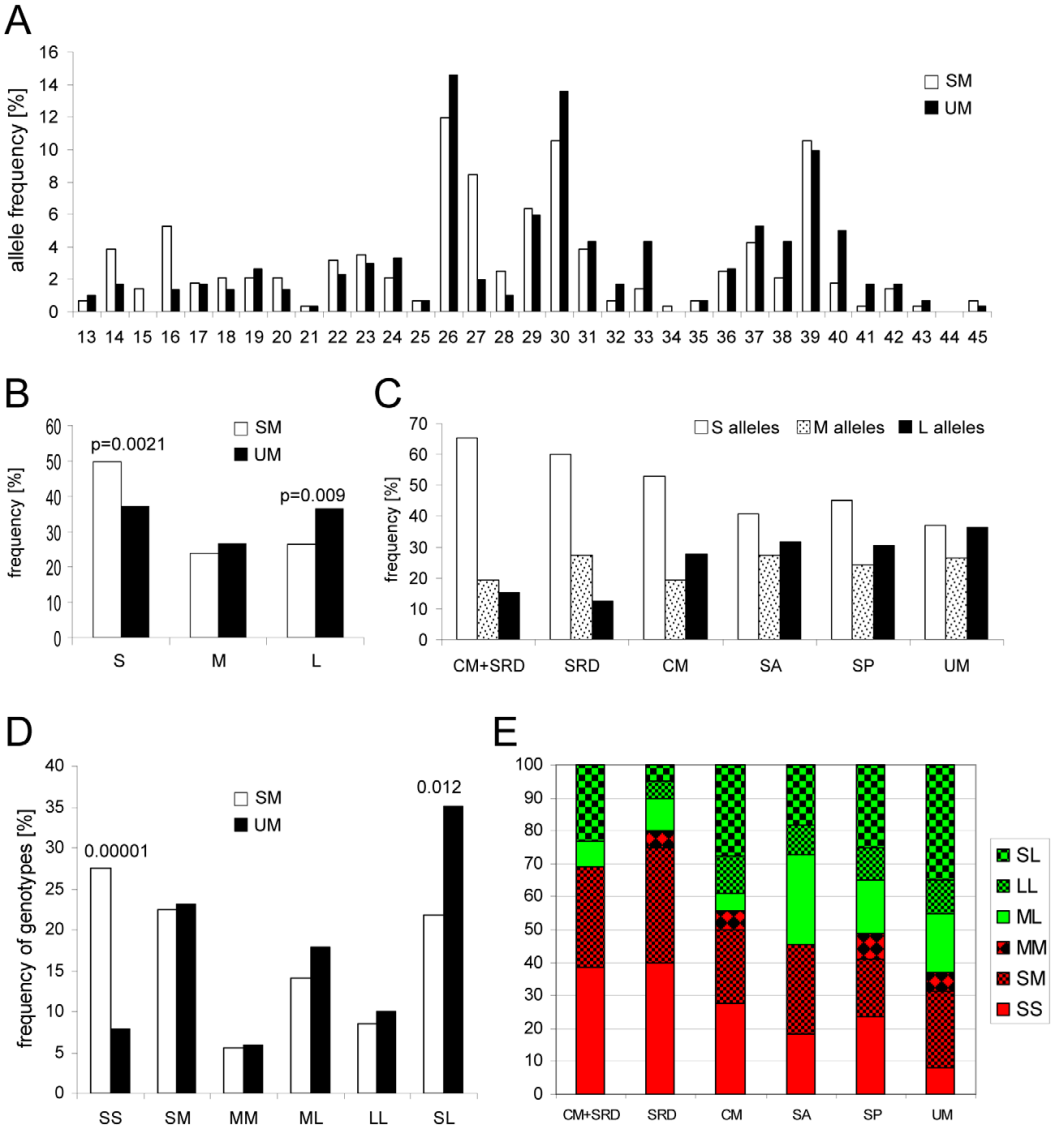
Long (GT)_n_ repeats and “L carrier” genotype associate with less severe disease. A) Frequency distribution of the number of [GT]_n_ repeats in SM (open bars, n = 282 alleles) and UM cases (black bars, n = 302 alleles). B) Frequency of the “S” (short<27), “M” (medium 27 to 32) and L (long>32) [GT]_n_ repeats for SM (open bars) and UM (black bars) cases. C) Frequency of “S” (open bars), “M” (dotted bars) and “L” (black bars) [GT]_n_ repeats according to disease entities. Distribution of *HMOX1* promoter genotypes amongst D) SM (open bars) and UM (black bars) cases, and E) amongst different disease entities. Those labeled in green are “L carriers”, those labeled in red are “non-L carriers”. SM = severe malaria, SRD = severe respiratory distress, CM = cerebral malaria, SA = severe anaemia, SP = severe prostration, UM = uncomplicated malaria.

In the study population (GT)_n_ variation ranged from 13 to 45 repeats, and the allele frequency distribution was trimodal with peaks at 26, 30 and 39 repeats (Figure 6A). Using a previously established classification [33], [34], [36], alleles were divided into 3 groups: “S” (<27 repeats), “M” (27 to 32 repeats), and “L” (>32 repeats). The frequency of “S” alleles was significantly higher in SM cases (0.50 vs. 0.37, p = 0.0021), whereas the frequency of “L” alleles was significantly higher in UM cases (0.36 vs 0.26, p = 0.009, Figure 6B). The frequency of different alleles according to disease entities is shown in Figure 6C.

Based on the “S-M-L” classification, six genotypes (SS, SM, MM, ML, LL and SL) were defined. As shown in Figure 6D, the “SS” genotype is significantly more frequent in SM cases compared to UM cases (27.5% vs. 8%, p<0.00001). Conversely, the “SL” genotype is more prevalent in UM cases (35% vs. 21.8%, p = 0.012). Figure 6E depicts the frequency of the 6 genotypes for each disease entity. When dichotomized as described previously [34], [56] into genotypes containing at least one “L” allele (LL, ML, SL = L-carriers; labeled green in Figure 6E) versus non-L carriers (SS, SM, MM; labeled red in Figure 6E), SM patients were 53% less likely to be L carriers than UM cases (OR: 0.47, CI 95%: 0.29 to 0.75, p = 0.002). When L carrier status was analysed in relation to different disease entities using multinomial logistic regression, cases with SRD and SRD plus CM were significantly less likely to be L carriers (85% and 74% less likely, respectively) compared to uncomplicated cases (Table 1). Of note, 9 out of the 10 individuals who succumbed to malaria were non-L carriers, compared to 53.4% non-L carriers within the remaining severe cases (p = 0.043, Fisher's exact test), or 37% non-L carriers within UM cases (p = 0.003, Fisher's exact test).

**Table 1:** Association between L allele containing genotype and different disease entities.

	SM (all entities)OR (95% CI)	p value	SPRR (95% CI)	p value	SARR (95% CI)	p value	CMRR (95% CI)	p value	SRDRR (95% CI)	p value	SRD+CM RR (95% CI)	p value
L carrier	0.47 (0.29–0.75)	0.002	0.62 (0.36–1.07)	0.087	0.71 (0.21–2.42)	0.582	0.47 (0.18–1.26)	0.135	0.15 (0.05–0.46)	0.001	0.26 (0.08–0.89)	0.032
	*0.49 (0.31–0.8)*	*0.004*	*0.66 (0.38–1.16)*	*0.148*	*0.61 (0.17–2.18)*	*0.446*	*0.48 (0.18–1.32)*	*0.155*	*0.16 (0.05–0.52)*	*0.002*	*0.28 (0.08–0.95)*	*0.042*

(SM = severe malaria, all entities, SP = severe prostration, SA = severe anaemia, CM = cerebral malaria, SRD = severe respiratory distress). In the top row, the odds ratio (OR) is shown for the association between L allele carriage and SM/UM, and a risk ratio (RR) is shown for the association between L allele carriage and each of the disease entities versus UM. (CI 95% refers to 95% confidence intervals of the estimates). The second row in italics shows similar results for the corresponding analyses adjusted for ethnicity.

We further examined whether ethnicity was associated with either disease outcome, frequency of L alleles or L allele containing genotypes. We found that ethnicity was not associated with being a SM or UM case (p = 0.294), or with any of the particular disease entities (p = 0.112). There was also no difference in the frequency of the L allele (p = 0.063) or of the L allele-containing genotypes (p = 0.088) among ethnic groups.

### The effect of *HMOX1* genotype on disease severity is not influenced by other factors associated with disease severity

To determine a possible confounding effect of some of the major factors known to determine disease severity, we measured the frequency of sickle cell trait, blood group O and G6PD deficiency, and the level of HRP-2 in our study population.

Hemoglobin S (HbS) confers protection from severe malaria in humans [57], and was recently shown to induce HO-1 in murine hematopoietic cells [58]. Blood group O has been associated repeatedly with reduced risk of severe malaria [59], and so has been G6PD deficiency [60], [61], as hypothesized by Allison [62]. In agreement with two previous studies using samples from this geographic area [37], [63], we confirmed the 968C/376G allele as the most common G6PD A- deficiency allele in our study population (6.26%). The latter study from the Gambia [37] suggested that heterozygous females and hemizygous males are relatively protected from severe disease. The histidine-rich-protein 2 (HRP-2) has been proposed as a surrogate for parasite biomass and is considered to be associated with disease severity [64]. Figure S3 shows the frequency of these factors according to disease group and *HMOX1* genotype. In our study population, carriage of the sickle cell trait, G6PDA^−^, or blood group O were neither associated with disease severity, nor with *HMOX1* genotype. As expected, HRP-2 was associated with disease severity, but showed no association with *HMOX1* genotypes.

### Higher *HMOX1* mRNA induction in non-L allele carriers

**Figure 7:**
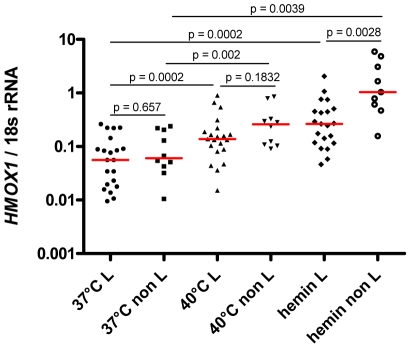
Higher *HMOX1* mRNA induction in peripheral blood from “non-L allele” carriers in response to hemin. Induction of *HMOX1* mRNA after 3 hrs exposure to 40°C or 100 µM hemin is shown according to L/non L carrier status for 26 individuals, using blood collected on D28. The red line represents the median. Raw p values are reported. After Bonferroni's adjustment for multiple comparisons the significance threshold becomes 0.007.

To examine whether the genotype of the (GT)_n_ polymorphism was associated with the magnitude of *HMOX1* mRNA induction in peripheral blood leucocytes in response to a defined stimulus, the data for hemin and heat-mediated induction of *HMOX1* mRNA in whole blood collected on D28 shown in Figure 3B were plotted according to L carrier status (Figure 7).

Both L and non L carriers had similar *HMOX1* expression at baseline (p = 0.65), and showed a significant 2.5 fold (L carriers, p = 0.0002), or 4.3 fold (non L carriers, p = 0.002) increase in *HMOX1* mRNA levels in response to heat. When hemin induced *HMOX1* mRNA levels were compared to baseline a 4.7 fold (L carriers, p = 0.0002), or 17.1 fold (non L carriers, p = 0.0039) increase was observed. Importantly, the median *HMOX1* mRNA measured in non-L carriers after hemin stimulation was 3.9 fold higher (p = 0.0028) than was observed in L carriers (Figure 7). In response to heat stimulation, non L carriers had 1.9 fold higher mRNA levels compared to L carriers (p = 0.183). After Bonferroni's adjustment for multiple comparisons the significance threshold for these tests becomes 0.007. The differential *HMOX1* mRNA expression of L and non L carriers in response to hemin is in line with what has been reported from human lymphoblastoid cell lines treated with H_2_O_2_ [33], and demonstrates that in our study population the magnitude of *HMOX1* mRNA expression in peripheral blood leucocytes in response to a defined stimulus is associated with the length of the (GT)_n_ repeat.

### Linkage disequilibrium around the *HMOX1* locus and population frequencies of L - alleles

To investigate the possibility that, in West Africans, the (GT)_n_ microsatellite is tightly linked to (or “tagging”) unexplored functional variants in alternative genes, we analyzed the extent of linkage disequilibrium (LD) at and around the *HMOX1* locus, using HapMap data for Nigerian Yoruba. In this West African population, the (GT)_n_ microsatellite in the promoter lies in a 6KB LD block that extends from rs2071746 (5′) to rs11912889 (3′), encompassing approximately half of the *HMOX1* gene. According to these data, the (GT)_n_ microsatellite would effectively tag only variants within *HMOX1*, but no other genes. Moreover, the two genes immediately flanking the *HMOX1* gene (*TOM1* 33 kb 5′ and *MCM5* 6 kb 3′ of *HMOX1*) are not obvious candidates for malaria susceptibility (Figure S4). Future comparative studies of LD would be ideally conducted in the Gambian ethnic groups, and will benefit from whole genome sequence data likely to emerge from initiatives including the 1000 Genomes Project [65].

The frequency of the long (>32 copy) repeat alleles was similar to that recently reported from Angola [30], but significantly higher than was reported from populations living in areas where malaria is not endemic, such as Europe (French [66], German [67]), and North America (Caucasians, [68]) or Asia (Japanese [34], [36], [69], Karen people from Myanmar [29]; see Table S2 in Text S1). The magnitude of the differences was therefore explored with fixation (*F*_ST_) indices. Differences between the Gambian and North American (*F*_ST_ = 0.29), or European populations (Gambia vs France, *F*_ST_ = 0.21; vs Germany, *F*_ST_ = 0.14) were slightly higher than the average for a genome-wide sample of polymorphisms (*F*_ST_ = 0.126, [70]), whereas differences between Gambian and Asian populations (*F*_ST_ for Gambia vs Myanmar, or vs Japan) were slightly less than the genome-wide average. In the Angolan population allele frequency of the long repeat alleles was higher than in The Gambia, and therefore slightly more divergent from the non-African population samples (Table S3 in Text S1). When available data from each continent were pooled, the largest *F*_ST_ values were observed for comparisons between Africa and America (*F*_ST_ = 0.33 CI 95%: 0.29 to 0.38), Africa and Europe (*F*_ST_ = 0.25 CI 95%: 0.2 to .29), and Africa and Asia (*F*_ST_ = 0.21 CI 95%: 0.18 to 0.25; Table S4 in Text S1).

## Discussion

Inspired by elegant studies in murine malaria models clearly demonstrating that the induction of HO-1 helps prevent severe forms of malaria [9], [10], and the intriguing possibility either to manipulate HO-1 activity pharmaceutically [71], [72] or to mimic its effect by administering CO [20], we explored the role of HO-1 in children with severe and uncomplicated *P. falciparum* infection. During acute disease, the number of WBC staining positive for HO-1, the *HMOX1* mRNA levels, and the HO-1 protein concentrations in plasma were significantly higher than during convalescence, being highest in the most seriously ill patients presenting with SRD.

While the association between elevated HO-1 and severe illness we and others [47], [68], [73] observed might merely reflect an appropriate response insufficient in magnitude and/or occurring too late, the association between short (GT)_n_ repeat alleles and increased inducibility of HO-1 *in vitro*, and more severe disease suggests that HO-1 levels above a certain threshold may be part of the causal pathway leading to severe disease and death. The association between short (GT)_n_ repeat alleles in the *HMOX1* gene promoter region (resulting in enhanced *HMOX1* mRNA expression) with CM observed in a small study in Myanmar [29], and more recently, in Angola [30] supports this notion. Intriguingly, both our study and the study carried out in Angola [30] observed a distinct peak around 39 (GT)_n_-repeats, which is in contrast to previous data from populations from non-malaria endemic areas [34], [36], [66], [67], [68], [69]. We have noted that the *F*_ST_ indices for comparisons between these two African populations and those from non-malaria endemic areas were slightly above the average for a genome-wide sample of polymorphisms [70], although more detailed study of polymorphism in this gene would be needed to test a neutral hypothesis. The possibility that the relatively high frequency of long (GT)_n_ repeats in Africa may have resulted from a survival advantage from *P. falciparum* should encourage investigation to prospect more powerfully for evidence of selection on this locus, given that malaria has been one of the most powerful selective forces acting on the human genome [74].

The fact that increased levels of indirect bilirubin and COHb – both end products of the reaction catalyzed by HO-1 – are widely recognized as independent markers for mortality in critically ill patients [23], [49], and that long (GT)_n_ alleles were associated with less frequent multi organ dysfunction in European ICU patients irrespective of the specific diagnosis [47], make it tempting to speculate that a high HO-1 response is disadvantageous for acute inflammatory conditions in general. In fact, HO-1-induced CO may reduce oxygen carrying capacity in the blood and tissue oxygenation, ultimately leading to metabolic acidosis. Furthermore, increased HO-1 may result in low nitric oxide (NO) levels [75]. This may constitute another pathway by which over-expression of HO-1 contributes to severe disease based on the beneficial effects ascribed to inhaled NO on endothelial function in patients with adult respiratory distress syndrome (ARDS) [76], and the accumulating evidence that depletion of NO contributes to severe malaria [77]. Considering that HO-1 overexpression in the liver leads to an increase in parasite liver load [11], and that the major benefit of RTS,S (a malaria vaccine that partially reduces the parasite burden in the liver) is the reduction of severe disease, it is tempting to speculate that particularly high HO-1 levels in the liver might contribute to severe malaria in man.

These findings differ from the role HO-1 plays in preventing severe disease in mice [8], [10]. An attempt to reconcile these observations needs to take into account that mice in contrast to humans lack the (GT)_n_ repeat polymorphism [18]. The functional relevance of the human (GT)_n_ promoter length polymorphism indicated here and elsewhere [33], [56] suggests that humans might have a greater genetically determined variability of *HMOX1* expression than exists among inbred mouse strains. The infection of BALB/c mice with *P. berghei* ANKA, for example, results in a fairly homogeneous 3–4 fold upregulation of *HMOX1* mRNA 6 days post infection [9], comparable to what we observed in uncomplicated cases. However, a more than 10 fold difference was measured in the most seriously ill patients of the SRD plus CM group. In fact, HO-1 has both pro-and anti-oxidant properties [24], and dependent on its amount, diametrically opposed effects have been described: Several *in vitro* studies have shown that moderate (less than 5 fold) induction of HO-1 is associated with protection against heme-mediated damage [78], while higher levels (greater than 10 fold) resulted in loss of cytoprotection [79]. Using HO-1 transfected hamster fibroblasts with either low, moderate, or high HO-1 activity, Suttner et al. demonstrated how HO-1 related cytoprotection turns into HO-1 mediated oxidative injury with increasing HO-1 expression [27]. Importantly, ferrous iron accumulated in high HO-1 expressing cells, and the addition of iron chelators or specific HO-1 inhibitors significantly reduced all measures of oxidative tissue injury [27]. The notion that high levels of HO-1 activity may potentiate, rather than attenuate ROS toxicity, and that this is related to the increased availability of ferrous iron is further supported by several in vitro studies [80], [81], as well as studies in animals [82], [83].

Thus, we hypothesize that, up to a certain level, induction of HO-1 is protective, while excessive upregulation of HO-1 in response to an inflammatory stimulus is deleterious.

The clinical relevance of free iron in severe malaria infections has been investigated previously, and high transferrin saturation (which indicates mobilization of ferrous iron) was associated with delayed recovery from coma in CM patients [84]. While HO-1 was not measured in this trial, a more recent study in patients with ARDS established that transferrin saturation increased in parallel to HO-1 [85], strengthening the idea that *in vivo* high HO-1 levels may result in a clinically relevant increase of ferrous iron. However, results of studies on the usefulness of iron chelation therapy with desferroxiamine in malaria patients have been inconclusive [86], [87].

We also provide mechanistic evidence that hemin-mediated *HMOX1* mRNA expression in neutrophils potentiates the magnitude of the neutrophil oxidative burst, and propose that a genetic predisposition to high levels of HO-1 may cause an otherwise protective response to become deleterious. By demonstrating how the neutrophil oxidative burst may influence disease severity, our data help to determine the role of this cell subset in the pathogenesis of severe malaria, which is currently ill-defined. Earlier *in vitro* studies established that iRBCs can be phagocytosed by neutrophils [88], and can activate them to produce ROS [89], which can kill parasites [50], [90]. In line with this, the amount of ROS produced by neutrophils from children with *P. falciparum* infection was associated with faster parasite clearance [91], and clinical protection from *P falciparum* correlated with neutrophil respiratory burst induced by merozoite antigens opsonized by antibodies [92], proposing neutrophils as an efficient defense mechanism. However, in murine models of severe malaria, early depletion of neutrophils prevents experimental CM [93], as well as sequestration of neutrophils to the lungs and reduces mortality [94], demonstrating that neutrophil effector mechanisms are capable of contributing to severe disease. Taken together, this suggests that an early neutrophil oxidative burst may benefit the host by contributing to initial parasite control, while a genetic predisposition towards an enhanced oxidative burst as suggested by our data may result in enhanced damage of endothelial cells, especially in conditions where neutrophils become sequestered in capillaries.

Consistent with our data, a genome-wide analysis of the host response to malaria recognized neutrophil-related gene expression responses as the principal pattern distinguishing convalescent from acute malaria patients, and the *HMOX1* gene was amongst the genes showing a stepwise increase with increasing severity [14].

The nature of the association between HO-1 in plasma and severe disease, and whether or not soluble HO-1 has a causal role in the pathogenesis of severe disease isn't entirely clear, but we consider it unlikely that HO-1 is functional in plasma. HO-1 is an intracellular enzyme [95], [96], and a molecule transporting HO-1 into the extracellular compartment has not been described. Furthermore, enzymatic functionality of HO-1 requires its C terminal end to be located in the membrane of the endoplasmic reticulum (ER) [97], and several electrons to be provided by an ER-bound NADPH cytochrome p-450 [98], [99]. Like Saukkonen et al. [47], we speculate that plasma HO-1 leaks from damaged tissue, and the association between plasma HO-1 and severe disease is primarily driven by the degree of tissue damage and not the degree of HO-1 induction itself. It is therefore not surprising that the *HMOX1* genotypes show no clear association with plasma HO-1 (Figure S5A). Based on the negative association we observed between plasma HO-1 and RBCs or Hb (both can be seen as markers of hemolysis in malaria), and considering that the release of free iron and heme-containing moieties that occurs during hemolysis leads to considerable damage of endothelial cells [48], [100], [101], we propose damaged endothelial cells as an important source of plasma HO-1.

This study had several limitations. In order to study HO-1 protein levels in WBCs according to disease entities or (GT)_n_ repeat polymorphisms, flow cytometric examination of blood from more participants would have been required.

In view of the results we obtained for the comparison of *in vitro* stimulated whole blood showing a significant difference in *HMOX1* mRNA expression between L and non L carriers in response to a defined amount of hemin (Figure 7), the lack of an association between *HMOX1* genotype and *HMOX1* mRNA expression *ex vivo* (Figure S5B) or the lack of a clear difference in *HMOX1* mRNA levels between SM and UM cases may be surprising. However, it is important to note that in contrast to the *in vitro* experiment where the nature and the strength of the stimulus is known, *in vivo HMOX1* mRNA expression may be driven by a variety of stimuli. To explore this further, it would have been necessary to measure various factors known to induce *HMOX1* mRNA expression *in vivo*. In this regard, our inability to measure free heme in plasma, known to be a major stimulus for HO-1, was an unforeseen limitation. We can therefore only speculate that inter-individual differences in the nature and amount of *HMOX1* mRNA inducing factors may have obscured the effect of the *HMOX1* promoter polymorphism on HO-1 levels *in vivo*.

With this caveat in mind, our data do indicate that a genetic factor affecting high HO-1 levels in response to heme is associated with more severe disease and death from malaria. We identified neutrophils as the predominant source of HO-1 in peripheral blood, provide evidence that increasing HMOX1 mRNA expression in these cells enhances the oxidative burst, and suggest that this may constitute a mechanism by which sequestered neutrophils cause tissue damage, thereby contributing to severe pathology. Considering that similar associations between high HO-1 and illness severity have been observed in other conditions [47], [73], limiting HO-1 activity pharmacologically with tin protoporphyrin IX [71] or other inhibitors may be an interesting therapeutic option worth considering.

An alternative therapeutic strategy might alter the distribution of HO-1 induction to particular cell types by therapeutic administration of haptoglobin or hemopexin, which both might limit the toxicity of free heme and restrict the uptake of cell free-hemoglobin and heme, and consequently upregulation of HO-1, to those cells bearing receptors for these molecules.

## Supporting Information

Figure S1 **Total numbers of HO-1 expressing leucocyte subsets.** The total numbers of cells that stained positive for HO-1 are shown for A) all white blood cells, B) neutrophils, C) monocytes, D) B cells, E) T cells and F) dendritic cells in blood from 14 severe (S) and 21 uncomplicated (U) cases. P values are derived from the random effects regression model, adjusting for age, gender, duration of symptoms and Hb. The red lines show the medians.
(TIF)

Figure S2 **Correlation between HO-1 and indirect bilirubin in plasma is shown for A) severe (n = 46, r: 0.69 p<0.0001) and B) uncomplicated malaria (n = 14, r: 0.77, p = 0.0012) cases.**
(TIF)

Figure S3 **Possible confounding factors.** The percentage of A) sickle cell trait carriers and B) children who are heterozygous (girls)or hemizygous (boys) carriers for the GdA^−^ variant of G 6PD deficiency are shown amongst severe (SM) or uncomplicated (UM) malaria cases, or amongst those classified as ‘L’ or ‘non-L carriers’ for the (GT)_n_ repeats. C) The percentage of children with blood group O is shown according to disease entity (left), or *HMOX1* promoter genotype (right). Error bars indicate the 95% CI for proportions, calculated using the Wilson method. P values are given for Fisher's exact test (A), or Chi Square test (B and C), respectively. D) Histidin rich protein-2 levels are shown according to disease entity (left), or *HMOX1* promoter genotype (right). The red line indicates the geometric mean. The p-value refers to regression analyses performed on log transformed data. (SP = severe prostration, SA = severe anaemia, CM = cerebral malaria, SRD = severe respiratory distress, UM = uncomplicated malaria).
(TIF)

Figure S4 **HapMap LD structure of the** ***HMOX1*** **locus.** The (GT)_n_ promoter polymorphism is located 138 bp upstream from rs2071746, at the left end of the 6 kb block. LD blocks are in the HaploView format, with both D' and r2 values shown.
(TIF)

Figure S5 **HO-1 plasma levels and** ***HMOX1*** **mRNA levels according to** ***HMOX1*** **promoter genotype.** A) HO-1 plasma levels and B) *HMOX1* mRNA levels are shown for *HMOX1* promoter genotypes, constituted of “S” (short<27), “M” (medium 27 to 32) and L (long>32) [GT]_n_ repeats alleles. The red line indicates the median.
(TIF)

Text S1 **Table S1 provides additional information on study participants according to clinical entities.** * IQR: Inter Quartile Range; ** weight-for-age z-scores for children up to the age of 10 years (10 years inclusive); WHO Growth Standards: For calculation of the z-scores the WHO packages “igrowup_stata” and “who2007_stata” for up to 5 years old and >6 to 10 year old children, respectively, were used. (http://www.who.int/childgrowth/software/en/ and http://www.who.int/growthref/tools/en/). (SP = severe prostration, SA = severe anaemia, CM = cerebral malaria, SRD = severe respiratory distress, UM = uncomplicated malaria). Table S2 shows an inter-population comparison for the HO-1 allele frequency analysis. The figures in bold print are the pooled data for each continent. Table S3 presents pairwise *F*_ST_ values for HO-1 length polymorphism allele frequency divergence between populations (>32 repeat alleles vs shorter alleles). The references of source data are in brackets. Table S4 presents pairwise *F*_ST_ values for HO-1 length polymorphism allele frequency divergence between continents (pooled data derived from table S2).
(DOC)
