# Supplementary figures and images for: HMOX1 Gene Promoter Alleles and High HO-1 Levels Are Associated with Severe Malaria in Gambian Children
Source: PLoS Pathog. 2012 Mar 15;8(3):e1002579. doi: 10.1371/journal.ppat.1002579 (PMC3305414; doi:10.1371/journal.ppat.1002579)

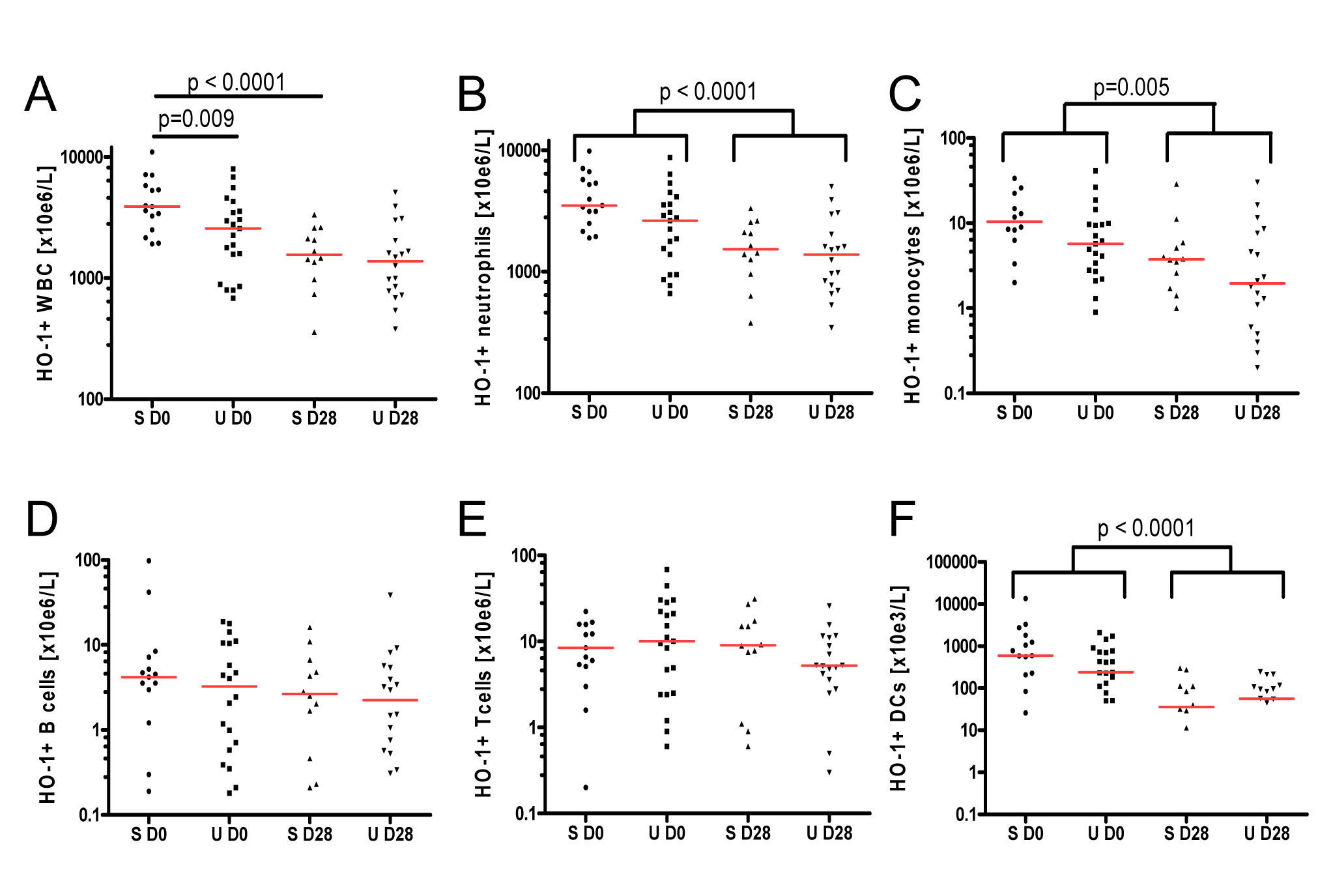

Supplement: Figure S1 — Total numbers of HO-1 expressing leucocyte subsets. The total numbers of cells that stained positive for HO-1 are shown for A) all white blood cells, B) neutrophils, C) monocytes, D) B cells, E) T cells and F) dendritic cells in blood from 14 severe (S) and 21 uncomplicated (U) cases. P values are derived from the random effects regression model, adjusting for age, gender, duration of symptoms and Hb. The red lines show the medians. (TIF) [file ppat.1002579.s001.tif]

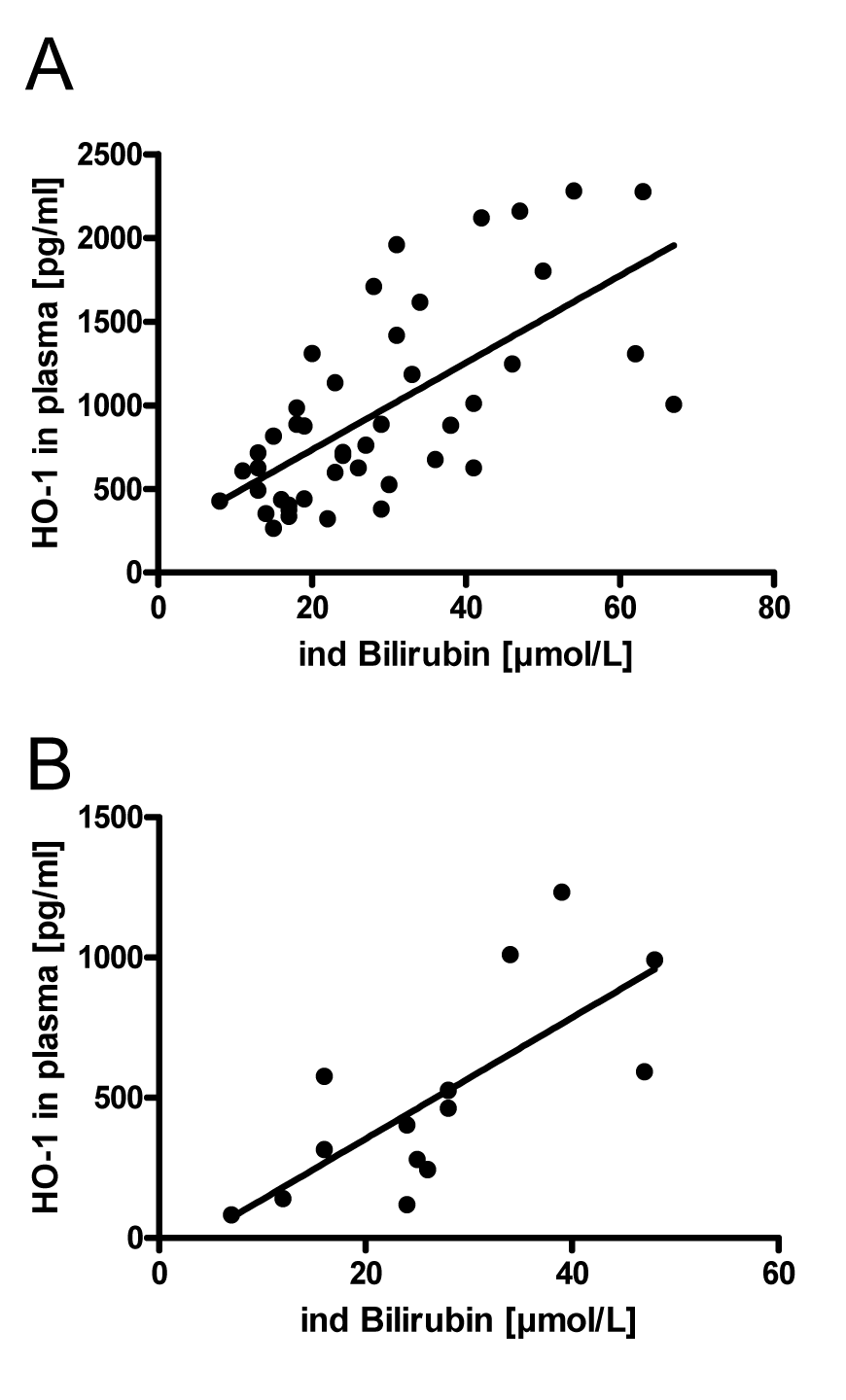

Supplement: Figure S2 — Correlation between HO-1 and indirect bilirubin in plasma is shown for A) severe (n = 46, r: 0.69 p<0.0001) and B) uncomplicated malaria (n = 14, r: 0.77, p = 0.0012) cases. (TIF) [file ppat.1002579.s002.tif]

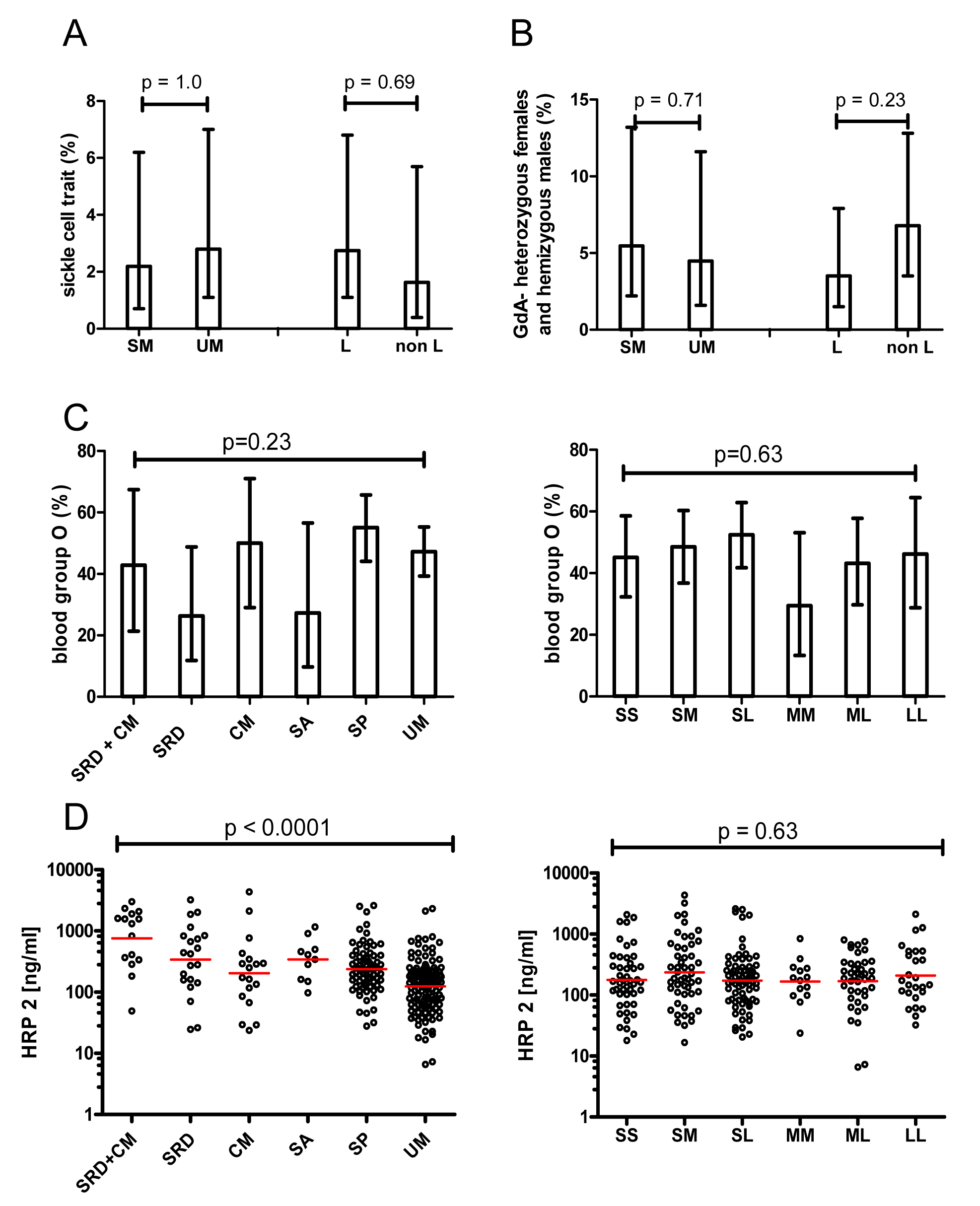

Supplement: Figure S3 — Possible confounding factors. The percentage of A) sickle cell trait carriers and B) children who are heterozygous (girls)or hemizygous (boys) carriers for the GdA− variant of G 6PD deficiency are shown amongst severe (SM) or uncomplicated (UM) malaria cases, or amongst those classified as ‘L’ or ‘non-L carriers’ for the (GT)n repeats. C) The percentage of children with blood group O is shown according to disease entity (left), or HMOX1 promoter genotype (right). Error bars indicate the 95% CI for proportions, calculated using the Wilson method. P values are given for Fisher's exact test (A), or Chi Square test (B and C), respectively. D) Histidin rich protein-2 levels are shown according to disease entity (left), or HMOX1 promoter genotype (right). The red line indicates the geometric mean. The p-value refers to regression analyses performed on log transformed data. (SP = severe prostration, SA = severe anaemia, CM = cerebral malaria, SRD = severe respiratory distress, UM = uncomplicated malaria). (TIF) [file ppat.1002579.s003.tif]

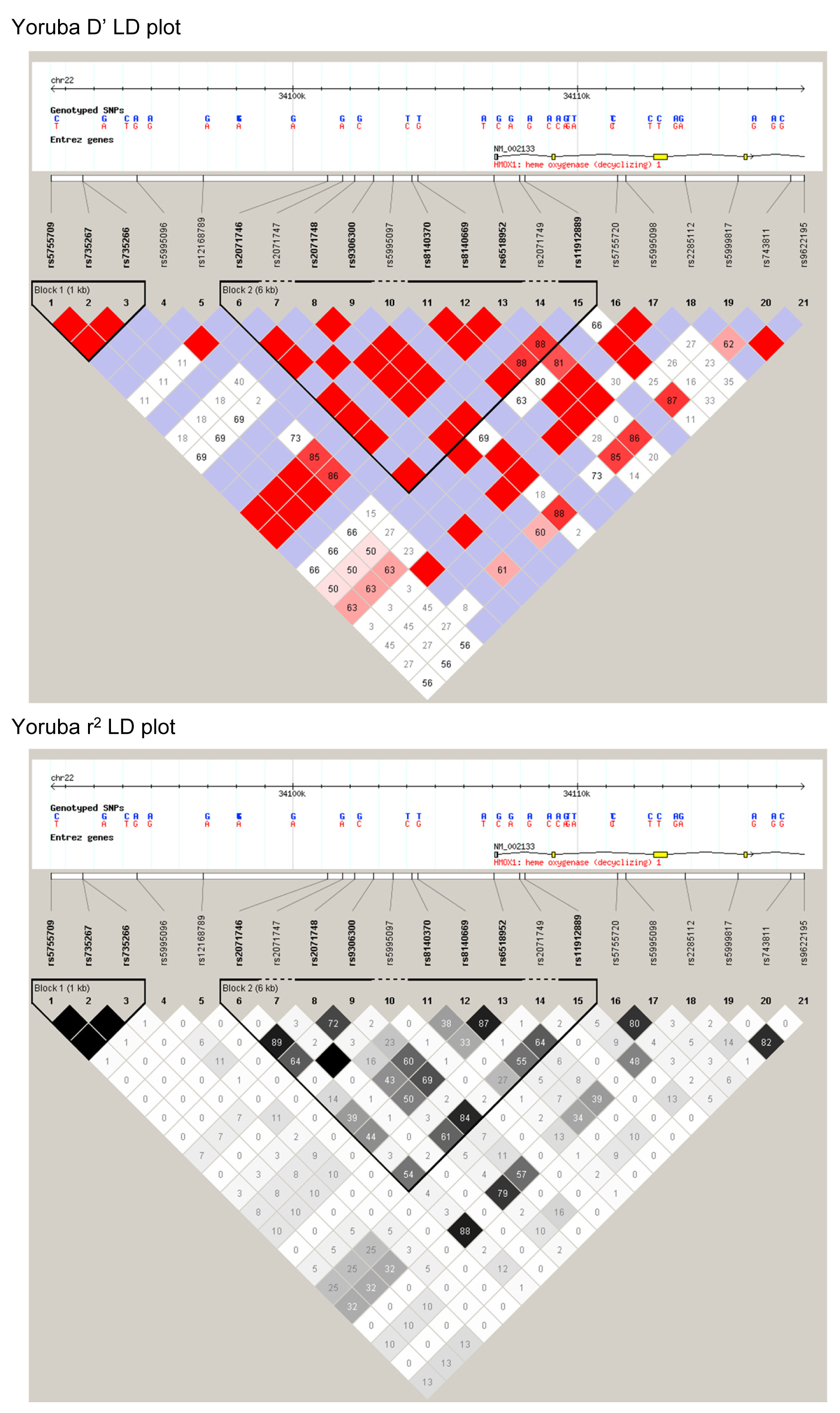

Supplement: Figure S4 — HapMap LD structure of the HMOX1 locus. The (GT)n promoter polymorphism is located 138 bp upstream from rs2071746, at the left end of the 6 kb block. LD blocks are in the HaploView format, with both D' and r2 values shown. (TIF) [file ppat.1002579.s004.tif]

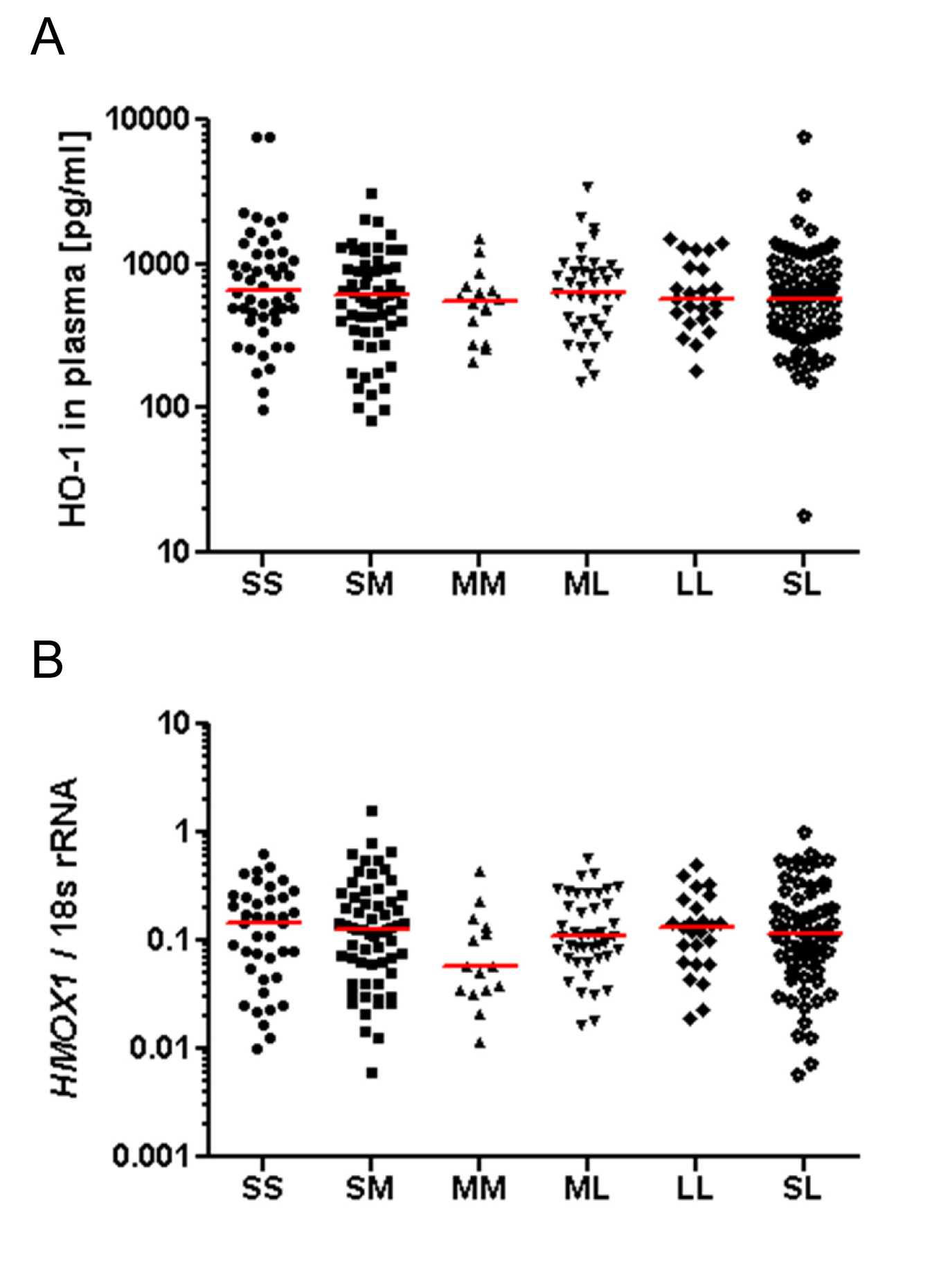

Supplement: Figure S5 — HO-1 plasma levels and HMOX1 mRNA levels according to HMOX1 promoter genotype. A) HO-1 plasma levels and B) HMOX1 mRNA levels are shown for HMOX1 promoter genotypes, constituted of “S” (short<27), “M” (medium 27 to 32) and L (long>32) [GT]n repeats alleles. The red line indicates the median. (TIF) [file ppat.1002579.s005.tif]
