# Supplementary material for: HMOX1 Gene Promoter Alleles and High HO-1 Levels Are Associated with Severe Malaria in Gambian Children
Source: PLoS Pathog. 2012 Mar 15;8(3):e1002579. doi: 10.1371/journal.ppat.1002579 (PMC3305414; doi:10.1371/journal.ppat.1002579)
Supplement: Text S1 — Table S1 provides additional information on study participants according to clinical entities. * IQR: Inter Quartile Range; ** weight-for-age z-scores for children up to the age of 10 years (10 years inclusive); WHO Growth Standards: For calculation of the z-scores the WHO packages “igrowup_stata” and “who2007_stata” for up to 5 years old and >6 to 10 year old children, respectively, were used. (http://www.who.int/childgrowth/software/en/ and http://www.who.int/growthref/tools/en/). (SP = severe prostration, SA = severe anaemia, CM = cerebral malaria, SRD = severe respiratory distress, UM = uncomplicated malaria). Table S2 shows an inter-population comparison for the HO-1 allele frequency analysis. The figures in bold print are the pooled data for each continent. Table S3 presents pairwise F ST values for HO-1 length polymorphism allele frequency divergence between populations (>32 repeat alleles vs shorter alleles). The references of source data are in brackets. Table S4 presents pairwise F ST values for HO-1 length polymorphism allele frequency divergence between continents (pooled data derived from table S2). (DOC) [file ppat.1002579.s006.doc]

**Table S1. Additional information on study participants according to clinical entities.**

| Severity group | Number enrolled | | Age (Day 0) | | Total WBC (Day 0) | | Total Neutrophil count (Day 0) | | Hb (Day 0) | | Parasitaemia (Day 0) | | z-score weight-for-age ** (Day 0) | | | Mortality (%)  (Day 0) | | Lactate (Day 0) | | Platelets (Day 0) |
| --- | --- | --- | --- | --- | --- | --- | --- | --- | --- | --- | --- | --- | --- | --- | --- | --- | --- | --- | --- | --- |
|  | n | % | median | IQR* | median | IQR* | median | IQR* | median | IQR* | Geometric mean | CI95% | n | median | IQR* | n | % | median | IQR* | Median and IQR* |
| SRD/CM | 17 | 5.5 | 4 | 4-5.6 | 8.9 | 6.4-10.2 | 4,074 | 3,051-6,880 | 9.2 | 7.8-10.4 | 448,114 | 281,431; 713,518 | 16 | -1.13 | -1.80 to  -0.34 | 5/17 | 29.4 | 10.1 | 8.3-12.5 | 38  (22-44) |
| SRD | 24 | 7.8 | 4 | 3-6 | 9.3 | 6.2-13 | 5,775 | 3,983-9,275 | 9.3 | 7.7-10.8 | 367,450 | 239,053; 564,810 | 21 | -0.83 | -1.82 to  -0.05 | 4/24 | 16.7 | 8.4 | 7.4-11 | 76  (29-139) |
| CM | 18 | 5.9 | 4.2 | 3-5 | 8.9 | 7.5-15 | 7,120 | 3,840-9,039 | 9.7 | 8.1-10.4 | 184,551 | 82,256; 414,062 | 18 | -0.89 | -1.71 to 0.21 | 0/18 | 0 | 4.4 | 3.8-5.2 | 65 (40-136) |
| SA | 11 | 3.6 | 2.6 | 1.8-4 | 20.2 | 13.7-23.2 | 9,004 | 8,300-9,926 | 4.9 | 4.1-5.8 | 282,288 | 192,298; 414,391 | 11 | -0.5 | -2.15 to 0.05 | 0/11 | 0 | 3.9 | 2.9-5.7 | 76 (45-95) |
| SP | 83 | 27 | 4.5 | 3-6 | 7.8 | 6-11.1 | 5,065 | 3,433-7,392 | 10.2 | 8.9-11.5 | 291,552 | 238,759; 356,018 | 77 | -1.32 | -2.15 to  -0.46 | 1/83 | 1.2 | 4.0 | 2.9-5.3 | 61 (39-126) |
| UM | 154 | 50.2 | 7 | 4.6-11 | 7.5 | 5.7-9.5 | 4,530 | 3,136-6,519 | 11.5 | 10.2-12.7 | 127,430 | 105,616; 153,749 | 110 | -0.9 | -1.8 to  -0.33 | 0/154 | 0 | 2.2 | 1.4-2.9 | 109  (66-161) |

***** IQR: Inter Quartile Range

** weight-for-age z-scores for children up to the age of 10 years (10 years inclusive);

WHO Growth Standards: For calculation of the z-scores the WHO packages “igrowup_stata” and “who2007_stata” for up to 5 years old and >6 to 10 year old children, respectively, were used. (<http://www.who.int/childgrowth/software/en/ and> <http://www.who.int/growthref/tools/en/>)
(SP = severe prostration, SA = severe anaemia, CM = cerebral malaria, SRD = severe respiratory distress, UM = uncomplicated malaria)

**Table S2.** Inter-population HO-1 allele frequency analysis

|  |  |  | no. of | > 32 repeat |  |
| --- | --- | --- | --- | --- | --- |
|  | population | characteristics | individuals | frequency | reference |
| Africa | Gambia | malaria cases | 293 | 0.316 |  |
|  | Angola | healthy controls | 211 | 0.450 | (30) |
|  |  |  | **504** | **0.372** |  |
|  |  |  |  |  |  |
| Europe | France | healthy controls | 749 | 0.075 | (66) |
|  | Germany | healthy controls | 301 | 0.093 | (67) |
|  |  |  | **1050** | **0.080** |  |
|  |  |  |  |  |  |
| Asia | Myanmar | malaria cases | 150 | 0.133 | (29) |
|  | Japan | healthy controls | 153 | 0.130 | (36) |
|  | Japan | healthy controls | 255 | 0.041 | (69) |
|  | Japan | healthy controls | 200 | 0.095 | (34) |
|  |  |  | **758** | **0.092** |  |
|  |  |  |  |  |  |
| America | North America | ICU patients | 1451 | 0.058 | (68) |
|  |  |  | **1451** | **0.058** |  |

(the figures in bold are the pooled data for each continent)

**Table S3.** Pairwise *F*_ST_ values for HO-1 length polymorphism allele frequency divergence between populations (> 32 repeat alleles vs shorter alleles)

|  | Gambia | Angola | France (66) | Germany (67) | Myanmar | Japan (36) | Japan (69) | Japan (34) | America (68) |
| --- | --- | --- | --- | --- | --- | --- | --- | --- | --- |
| Gambia |  |  |  |  |  |  |  |  |  |
| Angola | 0.034 |  |  |  |  |  |  |  |  |
| France | 0.206 | 0.393 |  |  |  |  |  |  |  |
| Germany | 0.14 | 0.293 | 0.001 |  |  |  |  |  |  |
| Myanmar(29) | 0.079 | 0.202 | 0.004 | 0.006 |  |  |  |  |  |
| Japan | 0.082 | 0.206 | 0.003 | 0 | 0 |  |  |  |  |
| Japan | 0.218 | 0.383 | 0.007 | 0.019 | 0.0566 | 0.053 |  |  |  |
| Japan | 0.128 | 0.270 | 0.001 | 0 | 0.0045 | 0.004 | 0.021 |  |  |
| America | 0.289 | 0.494 | 0.002 | 0.009 | 0.044 | 0.041 | 0.001 | 0.011 |  |

The references of source data are in brackets

**Table S4**. Pairwise *F*_ST_ values for HO-1 length polymorphism allele frequency divergence between continents (pooled data derived from supplementary table 2)

|  | Africa | Europe | Asia |
| --- | --- | --- | --- |
| Africa |  |  |  |
| Europe | 0.25 (0.20 to 0.29) |  |  |
| Asia | 0.21 (0.18 to 0.25) | 0.0003 (0 to 0.0059) |  |
| America | 0.33 (0.29 to 0.38) | 0.0036 (0.0002 to 0.0110) | 0.019 (0.0083 to 0.0325) |
